# Changes of saliva microbiota in the onset and after the treatment of diabetes in patients with periodontitis

**DOI:** 10.18632/aging.103399

**Published:** 2020-07-07

**Authors:** Ying Yang, Shili Liu, Yihua Wang, Zhibin Wang, Wenyu Ding, Xiaoyuan Sun, Kunlun He, Qiang Feng, Xiandang Zhang

**Affiliations:** 1 Endocrine and Metabolic Diseases Hospital of Shandong First Medical University, Shandong First Medical University & Shandong Academy of Medical Sciences, Jinan, Shandong, China; 2 Department of Human Microbiome, School and Hospital of Stomatology, Cheeloo College of Medicine, Shandong University, Jinan, Shandong, China; 3 Shandong Provincial Key Laboratory of Oral Tissue Regeneration and Shandong Engineering Laboratory for Dental Materials and Oral Tissue Regeneration, Jinan, Shandong, China; 4 School of Basic Medical Science, Cheeloo College of Medicine Shandong University, Jinan, Shandong, China; 5 Shandong University Hospital, Jinan, Shandong, China; 6 Beijing Key Laboratory for Precision Medicine of Chronic Heart Failure, Chinese PLA General Hospital, Beijing, China; 7 State Key Laboratory of Microbial Technology, Shandong University, Qingdao, Shandong, China

**Keywords:** T2DM, salivary microbiota, treatment, differential bacteria, microbial markers

## Abstract

The relationship between type 2 diabetes mellitus (T2DM) and oral microbiota is still insufficiently recognized. In the present study, we compared the salivary microbiome of nondiabetic individuals, treatment-naïve diabetic patients, and diabetic patients treated with metformin or a combination of insulin and other drugs. The α- and β-diversity demonstrated significant differences in the salivary microbiome between the nondiabetic people and patients with a history of diabetes, while little divergence was found among individuals with a history of diabetes. After characterizing the effects of periodontitis on the microbial composition of each group, the salivary microbiome of the treatment-naïve diabetic patient group was compared with that of nondiabetic people and the metformin/combined treatment groups. The results revealed changes in the contents of certain bacteria after both the onset and the treatment of diabetes; among these differential bacteria, *Blautia_wexlerae, Lactobacillus_fermentum, Nocardia_coeliaca and Selenomonas_artemidis* varied in all processes. A subsequent correlational analysis of the differential bacteria and clinical characteristics demonstrated that salivary microbes were related to drug treatment and certain pathological changes. Finally, the four common differential bacteria were employed for distinguishing the treatment-naïve diabetic patients from the nondiabetic people and the treated patients, with prediction accuracies of 83.3%, 75% and 75%, respectively.

## INTRODUCTION

Type 2 diabetes (T2D) is an important health problem for more than 380 million people worldwide [1], and the International Diabetes Federation (IDF) estimates that the prevalence of T2D will exceed 10% among the global adult population by 2040 [2]. T2D is closely correlated with the abnormality of the gut microbiota, treatment of mice with Enterobacter cloacae B29 isolated from the intestines of diabetic patients led to obesity and insulin resistance [3]. Subcutaneous injection of *E. coli* endotoxin triggered insulin resistance and obesity [4]; On the contrary, intervening intestinal flora through effective means may present a novel ecological approach for managing T2DM, such as targeted restoration of SCFA producers [5], or modulating bacteria-mucosal immunity-inflammation [6]. A variety of mechanisms have been proposed between gut microbes and T2D development. For example, the alteration of the gut microbiome in T2D patients may promote intestinal permeability, reduce butyrate production and increase lipopolysaccharide (LPS) production, while LPS levels could trigger significant increases in plasma glucose levels and insulin resistance [7, 8]. In addition, changes in the gut microbiota in T2D patients could alter energy homeostasis, modulate intestinal barrier integrity, change gastrointestinal peptide hormone secretion, promote fat accumulation and modulate host inflammatory status [9, 10]. Given that dysbiosis tends to occur earlier than clinical characteristics and is more sensitive to treatment, the microbiota is possibly of importance for the clinical diagnosis and treatment of T2D [11].

The most commonly used drugs for T2D treatment are metformin, and the well-known mechanism of metformin is to activate hepatic AMP-activated protein kinase (AMPK) and reduce liver gluconeogenesis by inhibiting mitochondrial glycerophosphate dehydrogenase [12]. While studies showed that the beneficial effects of metformin may be mediated by intestinal microbes [13, 14], metformin treatment increased the abundance of mucin-degrading bacteria and short-chain-fatty-acid–producing microbes [15, 16] and promoted the gut microbiota to produce *bsh*, a gene encoding a bile salt hydrolase, which was significantly negatively correlated with the percentage of glycated hemoglobin [17]. Another commonly used medication for diabetes, insulin, could also increase the proliferation of nonbutyrate-producing bacteria, while butyrate-producing bacteria in the gut, such as *Roseburia intestinalis* and *Eubacterium hallii*, could improve insulin sensitivity [18, 19].

Compared with healthy individuals, patients with T2D presented significant dissimilarities in oral microbiota biodiversity [20], such as decreasing *Bifidobacterium* and increasing *Streptococcus* and *Lactobacillus* [21]. Mice fed a diabetogenic diet developed gingival inflammation and alveolar bone loss and increased prevalence of periodontal pathogenic microbes in the oral cavity, such as *Fusobacterium nucleatum and Prevotella intermedia* [22]*.* The multiple bacterial taxa in the phylum Actinobacteria were associated with the risk of T2D [23], and the periodontal pathogens, such as *Porphyromonas gingivalis* and *Aggregatibacter actinomycetemcomitans*, were correlated with diabetes risk and glycemic control [24, 25]. Moreover, diabetes enhanced the expression of IL-17, which altered the composition of oral microbiota, reduced the secretion of IL-6 and RANKL and inhibited neutrophil recruitment and bone resorption [26].

The salivary microbiome reflects the whole oral microecosystem, and the analysis of the salivary microbiome could reflect alterations in the oral microbiome and the development of diabetes. In this study, we applied 16S rRNA sequencing to detect changes in the salivary microbiome from healthy, treatment-naïve diabetic and diabetic treated individuals. Our results revealed dramatic changes in the salivary microbiome at the onset and during the treatment of diabetes. This study enables us to further understand the relationship between the oral microbiota and T2D.

## RESULTS

### Grouping information and the comparison of α- and β-diversity

We collected 102 salivary samples from four cohorts, namely, nondiabetic people (Group A, 32 samples), treatment-naïve T2D patients (Group B, 31 samples), T2D patients with metformin treatment (Group C, 17 samples), and T2D patients with combined medication treatment (Group D, 22 samples, insulin plus metformin or other hypoglycemic drugs) (Supplementary Tables 1 and 2). As T2D patients are often troubled with periodontal disease, the oral healthy states were also considered and recorded in detail. All samples were analyzed by 16S rRNA sequencing; detailed information was introduced in the Materials and Methods section. After quality filtering, more than 8.09 million clean reads were harvested, corresponding to a mean of 79,314 effective tags and 211 OTUs per sample (Supplementary Tables 3 and 4). The treatment-naïve diabetic patient group had a similar number of clean reads (Group A: 80816±2810; Group B: 77559±7542; Group C: 78826±6623; Group D: 80892±3417) to the nondiabetic people and the diabetic patients after treatments (p>0.05). The rarefaction curve demonstrated that all samples tended to be saturated (Supplementary Figure 1), suggesting that the OTUs covered most of the bacterial species that exist in the saliva.

**Figure 1:**
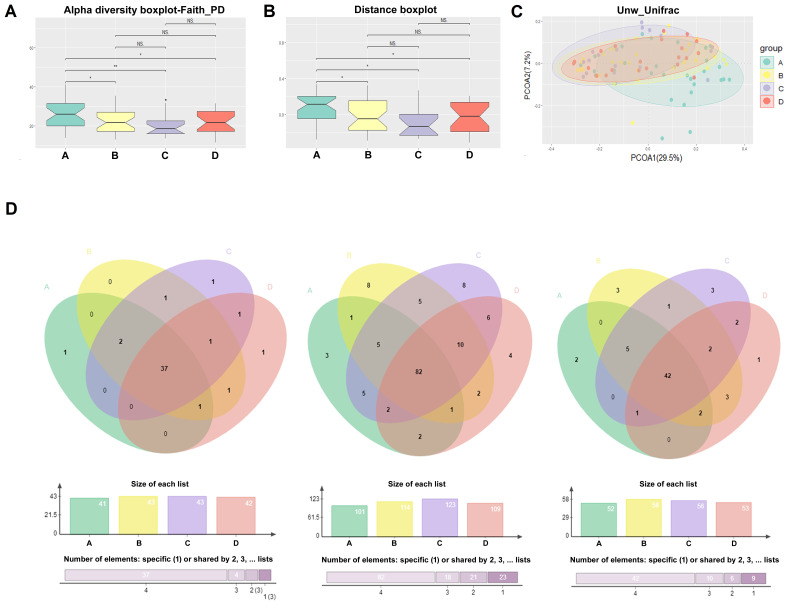
**Comparison of alpha diversity and beta diversity.** The α-diversity (**A**), β-diversity (**B**), and the principal coordinate analysis (PCoA) of the Unweighted Unifrac distance (**C**) results of the salivary microbiota among the four groups; (**D**) The salivary “core microbiome” shared by each group at the genus, OTU and species level, respectively.

The α-diversity analysis of the salivary microbiota among the four groups showed that the α-diversity indexes of nondiabetic people (group A) were significantly higher than patients with a history of T2D (group B, C and D) (P value < 0.05) (Figure 1A). However, the diversity of the salivary microbiota did not change significantly after metformin or combined treatment (Figure 1A). The number of OTUs identified in nondiabetic people was significantly higher than that identified in treatment-naïve diabetic patients (p=0.0392) and metformin-treated diabetic patients (p=0.0001). On average, 233, 207, 181 and 208 OTUs were obtained from the salivary microbiota of Groups A, B, C and D, respectively (Supplementary Figure 2). The β-diversity results showed the differences between nondiabetic people and patients with a history of diabetes (Figure 1B). The principal coordinate analysis (PCoA) of the unweighted UniFrac distance showed that the distribution of salivary microbiota that existed in nondiabetic individuals was more dispersed than that in individuals with a history of diabetes (Figure 1C), while the index was similar in the treatment-naïve diabetic and metformin/combined medication groups.

The salivary “core microbiome” shared by each group was also analyzed at the genus, OTU and species level, respectively. On the genus level, 41, 43, 43, and 42 genera were shared by the individuals of Groups A, B, C and D, respectively (Figure 1D and Supplementary Figure 3 and 4); on the OTU level, the numbers were 101, 114, 123 and 109, respectively (Figure 1D and Supplementary Figure 5 and 6); and at the species level, the numbers were 52, 58, 56 and 53, respectively (Figure 1D). In addition, 37 genera, 82 OTUs and 42 species were shared among all four groups (Figure 1D). The salivary “core microbiome” covers the majority of bacteria at different taxonomic levels in each group; therefore, the abundance of salivary bacteria in the “core microbiome” varied significantly during the onset and treatment of diabetes.

### The study of salivary microbiota changes and periodontitis

**Figure 2:**
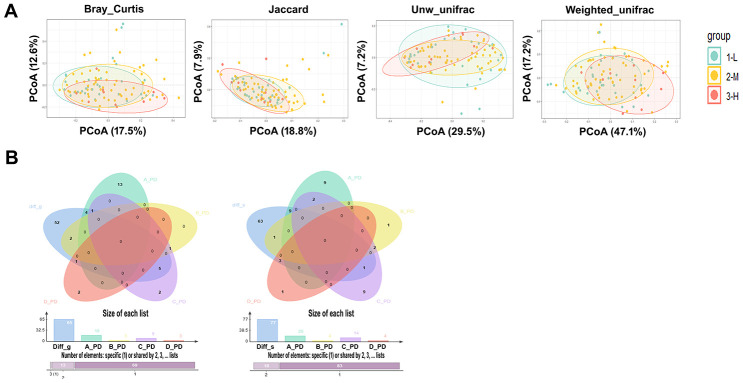
**The study of salivary microbiota changes and periodontitis.** (**A**) PCoA results of the salivary microbiota calculated by the severity of different periodontitis in all individuals, the p values were obtained by ADONIS (permutational MANOVA) analysis with Bray Curtis, Jaccard, unweighted and Weighted Unifracs. (**B**) The bacterial genera (Left) and species (Right) with significant changes in L, M, and H stage of periodontitis in healthy individuals were not included as the differential genera and species in the onset and treatment of diabetes, and the p value showed no significant difference when these differential genera and species were used to compare the naïve diabetic group with the other three groups.

As the differences in the severity of periodontitis in each group may lead to changes in the oral microbiome between groups, we first examined the differences in the severity of periodontitis between groups. The severity of periodontitis was elevated in the individuals with treatment-naïve diabetes in comparison to nondiabetic people, and treatment with metformin, insulin or some other hypoglycemic drugs did not alleviate the severity of periodontitis. The PCoA results of the salivary microbiota calculated by the differences in the severity of periodontitis in all individuals showed no significant differences in β-diversity in mild (L), moderate (M), and severe (H) periodontitis (Figure 2A); the p value obtained by the adonis function (permutational MANOVA) analysis of the periodontitis-associated bacteria was 0.45, 0.15, 0.65 and 0.24, respectively, for Groups A, B, C and D, suggesting that the change in the β-diversity of the salivary microbiota between different T2D groups was not related to the severity of periodontitis (Supplementary Figure 7).

In addition, microbial changes due to periodontitis within Group A were analyzed, and a variety of periodontitis-related pathogens, such as *Porphyromonas_gingivalis and* *Parvimonas_micra*, were increased with the severity increase of periodontitis (Supplementary Table 5). However, the number of microbes that correlated with the severity of periodontitis within the T2D groups (Groups B, C and D) was reduced after the onset of diabetes. Noticeably, the periodontitis pathogen *Porphyromonas_gingivalis* was not significantly increased with the severity of periodontitis in individuals with a history of T2D. Moreover, the bacterial genera and species with significant changes in the L, M, and H stages of periodontitis in nondiabetic individuals were not included as the differential genera and species in the onset and treatment of diabetes (Figure 2B); these differential genera and species were used to compare the treatment-naïve diabetic group with the other three groups, and the p value showed a significant difference. The above results demonstrate that the bacterial difference between T2D groups is not significantly influenced by periodontitis and that T2D may alter the salivary microbiome caused by periodontitis.

### Changes in the salivary microbiota under the conditions of diabetes and its treatments

**Figure 3:**
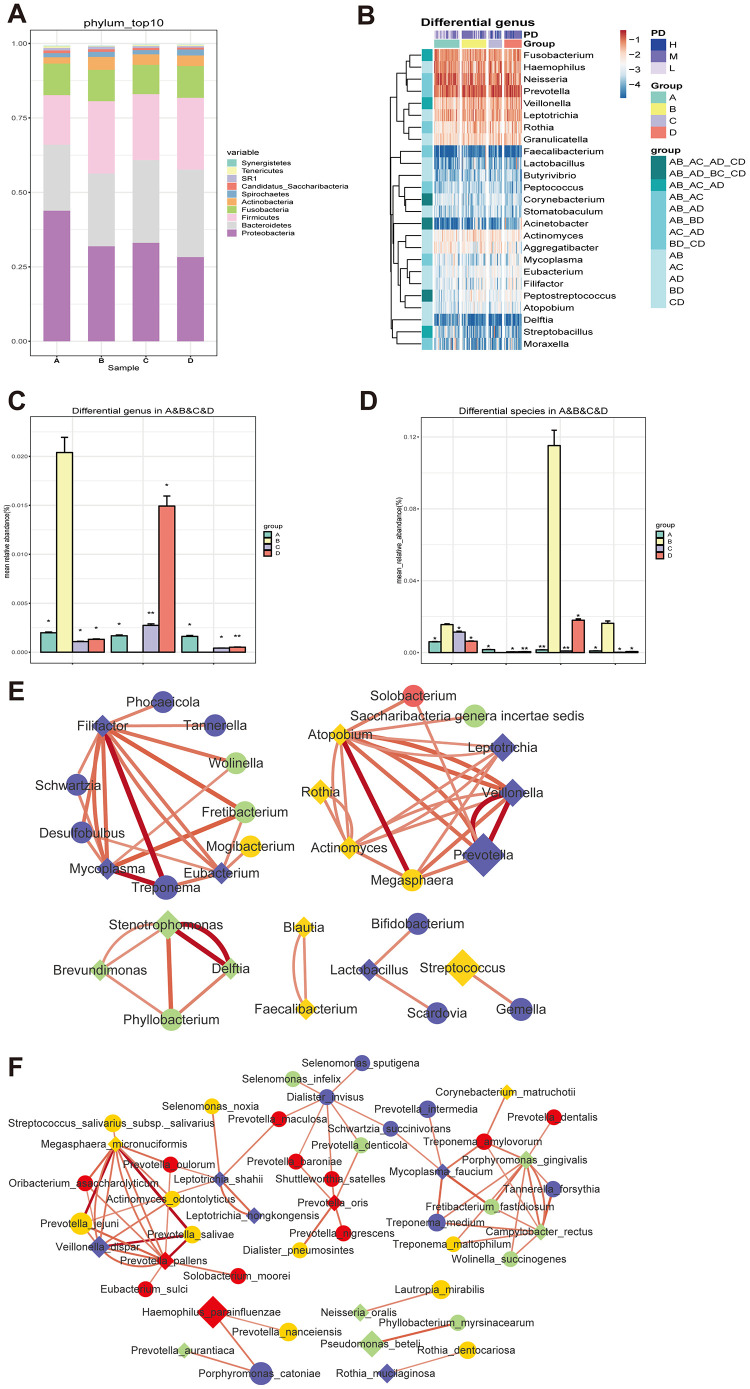
**Changes of the salivary microbiota under the circumstance of diabetes and treatments.** (**A** and **B**) The salivary microbiome in healthy people (Group A) and naïve diabetic patients (Group B) as well as diabetes treated with metformin (Group C) and combined medication (Group D) were compared at the phylum level; (**C** and **D**) the amount of three genera (Blautia, Cobetia and Nocardia) and four species (*Blautia_wexlerae, Lactobacillus_fermentum, Nocardia_coeliaca and Selenomonas_artemidis*) with relatively high abundance in the naïve diabetic patients were significantly different from both healthy people and the treatment groups; (**E** and **F**) |Spearman correlation| ≥0.7 and q value≤0.01 analysis of the salivary microbiota at the genus and species levels with the abundance ≥ 0.02%.

To clarify the effects of diabetes on salivary microbiota, we first compared the salivary microbiome in nondiabetic people (Group A) and in treatment-naïve diabetic patients (Group B) at the phylum, genus and species levels. At the phylum level, Proteobacteria was the most abundant phylum in nondiabetic people, covering more than 40% of the salivary bacterial mass, while it was significantly reduced in treatment-naïve diabetic patients (Figure 3A). In contrast, Bacteroidetes, Firmicutes and Fusobacteria were increased significantly in Group B. At the genus level, 27 genera showed significant changes between the two groups (Supplementary Tables 6 and 7). *Prevotella* was the most abundant genus among the salivary microbiota of Group B, covering approximately 15% of the whole salivary bacterial mass (Supplementary Figure 8), while its abundance was lower than that of the genera *Neisseria* and *Haemophilus* in Group A (Supplementary Figure 8). At the species level, a total of 37 species changed significantly in Group B in comparison to Group A (Supplementary Tables 6 and 7), such as *Prevotella_aurantiaca, Prevotella_oris and Streptococcus_mutans*. *Haemophilus parainfluenzae* was the most abundant bacteria in both groups (Supplementary Figure 8), but it was not significantly different between the two groups. *Pseudomonas_beteli* showed a clear change between Groups A and B (Supplementary Figure 8 and Supplementary Tables 6 and 7).

To evaluate the effect of diabetes treatment on salivary microbiota, we explored the bacterial difference in treatment-naïve diabetic (Group B) and metformin-treated individuals (Group C) at the phylum, genus and species levels. At the phylum level, Fusobacteria was reduced after metformin treatment (Figure 3A). At the genus level, 11 genera showed significant changes between Group B and Group C (Supplementary Figure 8 and Supplementary Tables 6 and 7), including *Corynebacterium*, *Blautia*, *Nocardia*, *Lactococcus*, etc. At the species level, a total of 18 species differed significantly (Supplementary Figure 8 and Supplementary Tables 6 and 7), such as *Corynebacterium_matruchotii, Lactobacillus_animalis* and *Roseburia_inulinivorans*.

**Scheme 1:**
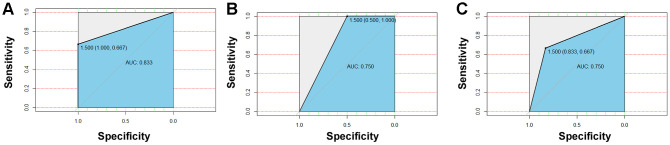
**Summary of the differences between saliva microbiota of healthy people (Group A), naïve diabetic patients (Group B), diabetes treated with metformin (Group C) and combined medication (Group D).**

We further compared the salivary microbiome changes between treatment-naïve diabetic patients (Group B) and diabetes patients treated with combined medication (Group D). At the phylum level, Proteobacteria were decreased and were Bacteroidetes increased significantly in Group D (Figure 3A). At the genus level, 15 genera showed significant changes in T2D patients with combined medication treatment (Supplementary Figure 8 and Supplementary Tables 6 and 7), and the ratio of the relatively abundant genera *Neisseria*, *Haemophilu*s and *Streptococcus* remained unchanged. At the species level, a total of 19 microbes changed significantly in Group D (Supplementary Figure 8 and Supplementary Tables 6 and 7), such as *Faecalibacterium_prausnitzii, Lactobacillus_iners* and *Streptococcus_sobrinus*. The above results are summarized in Scheme 1.

The bacterial genera and species that showed significant differences are illustrated in Figure 3B and Supplementary Figure 9, respectively. Noticeably, the amount of three genera (*Blautia*, *Cobetia* and *Nocardia*) and four species (*Blautia_wexlerae, Lactobacillus_fermentum, Nocardia_coeliaca and Selenomonas_artemidis*) with relatively high abundance in the treatment-naïve diabetic patients were significantly different from that of both nondiabetic people and the treatment groups (Figure 3C and 3D, Supplementary Tables 6 and 7).

To study the correlation between the differently distributed bacteria among the four groups, |Spearman correlation| ≥0.7 and q value≤0.01 were employed to examine the salivary microbiota at the genus and species levels with an abundance ≥0.02%. The results showed that there were close correlations within groups at the genus level, and clear differences in the bacterial correlation numbers were observed among the four groups (Figure 3E). The bacterial interactions at the species level were similar to those at the genus level (Figure 3F), indicating the interdependence of bacteria in the salivary microbiota and the change in their correlation under the conditions of treatment-naïve diabetes and drug treatments.

### Correlations between salivary bacteria and clinical parameters

**Figure 4:**
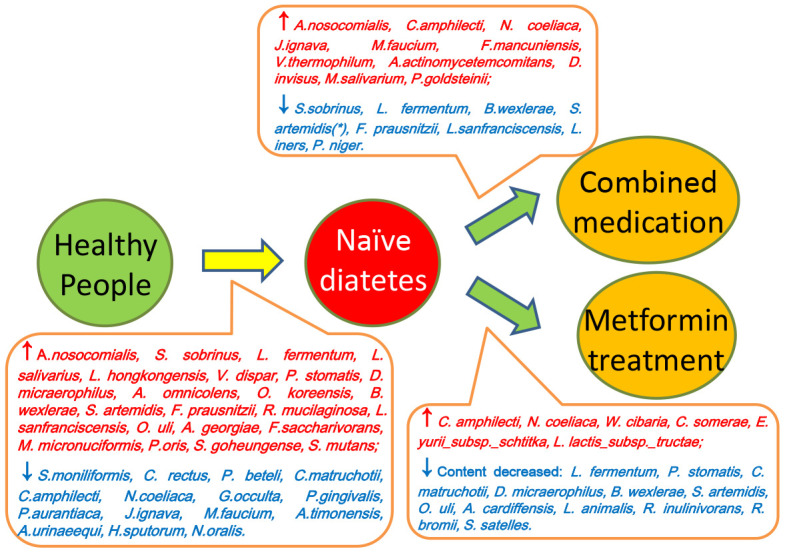
**Correlations between salivary bacteria and clinical parameters.** |Spearman correlation| ≥0.7 and q value≤0.01 analysis of the salivary microbiota at the genus (**A**) and species (**B**) levels with the abundance ≥ 0.02%.

The correlations between differentially distributed bacteria in each group and various clinical characteristics (Supplementary Tables 1 and 2) were analyzed by Spearman correlation analysis (“Hmisc” in R package), species and clinical parameters with p adj<=0.05, ǀcorǀ >=0.3 were selected. The results showed that some differential genera (Figure 4A and Supplementary Figure 10) and species (Figure 4B and Supplementary Figure 11) were related to clinical characteristics, such as fasting blood sugar (Glu), body mass index (BMI), periodontitis (PD), blood urea nitrogen (BUN), systolic blood pressure (SBP), total cholesterol (TCHO), glycosylated serum protein (GA.L), creatinine (Cre) and alanine aminotransferase (ALT). These characteristics showed that abnormalities in the salivary microbiome were closely correlated with diabetes, such as the blood glucose level and parameters used to assess the effects of treatment. In addition, in the salivary microbiota of nondiabetic people, SR1_genera_*Incertae_sedis* was related to blood glucose levels at the genus level; *Capnocytophaga*, *Moraxella*, *Filifactor* and *Abiotrophia* were related to BMI; 14 genera, such as *Porphyromonas*, *Treponema*, *Catonella* and *Tannerella*, were related to PD; 11 genera, such as *Prevotella*, *Veillonella* and *Alloprevotella*, were related to sex; and *Moraxella* was related to age. This condition was changed in treatment-naïve T2DM patients, *Abiotrophia* was related to BUN, *Veillonella* was related to PD, *Lautropia* was related to ALT, and *Wolinella* was related to smoking or drinking. At the species level, 12 species, such as *Veillonella_dispar, Rothia_mucilaginosa* and *Prevotella_jejuni*, were positively related to sex In healthy control saliva, 16 species, *Porphyromonas_gingivalis, Campylobacter_rectus, Neisseria_oralis,* and *Granulicatella_elegans*, were positively related to PD; *Porphyromonas_gingivalis* and *Cardiobacterium_valvarum* were positively related to age; *Abiotrophia_defectiva* was positively related to BMI; and *Oribacterium_asaccharolyticum* was positively related to GLU. In patients with a history of diabetes, *Leptotrichia_hofstadii* was positively related to sex; *Wolinella_succinogenes* was positively related to smoking and drinking; *Veillonella_dispar* was positively related to PD; *Haemophilus_sputorum* was positively related to SBP; *Prevotella_shahii* was positively related to GA.L; *Lautropia_mirabilis* was positively related to ALT; *Neisseria_oralis* was positively related to BUN; *Abiotrophia_defectiva* was positively related to BUN; *Granulicatella_elegans* and *Alloprevotella_rava* was positively related to CRE; and *Cardiobacterium_hominis* was positively related to TCHO. The above results suggest a close connection between salivary bacteria and the clinical characteristics of diabetes.

### Detection and testing of salivary microbial markers

To study the effect of sex and age of the subject as well as the number of OTUs on the composition of the oral bacterial community, we grouped the samples according to their sex, age, and OTUs, and ADONIS (also known as permutational MANOVA) was employed to analyze the contribution of different grouping factors to sample differences, and use the Permutation test for statistical significance p value and R2). According to the results of PERMANOVA analysis, only the OTUs of each sample has a significant effect on the composition of saliva bacterial community (P< 0.05) (Supplementary Table. 8). Next, we did a correlation analysis between the microbiota and OTUs to find out whether the bacteria that have the larger correlation with OTUs coincide with the biomarker we found earlier. The results showed that 22 species were obtained when p<= 0.05, the absolute value of the correlation coefficient |cor|> = 0.3 (Supplementary Table. 9). However, the bacteria we choose as biomarker, *Blautia_wexlerae, Lactobacillus_fermentum, Nocardia_coeliaca and Selenomonas_artemidis*, do not intersect with these 22 species of bacteria (Supplementary Figure 12), so the biomarkers we found in this study are not affected by the upward factors.

**Figure 5:**
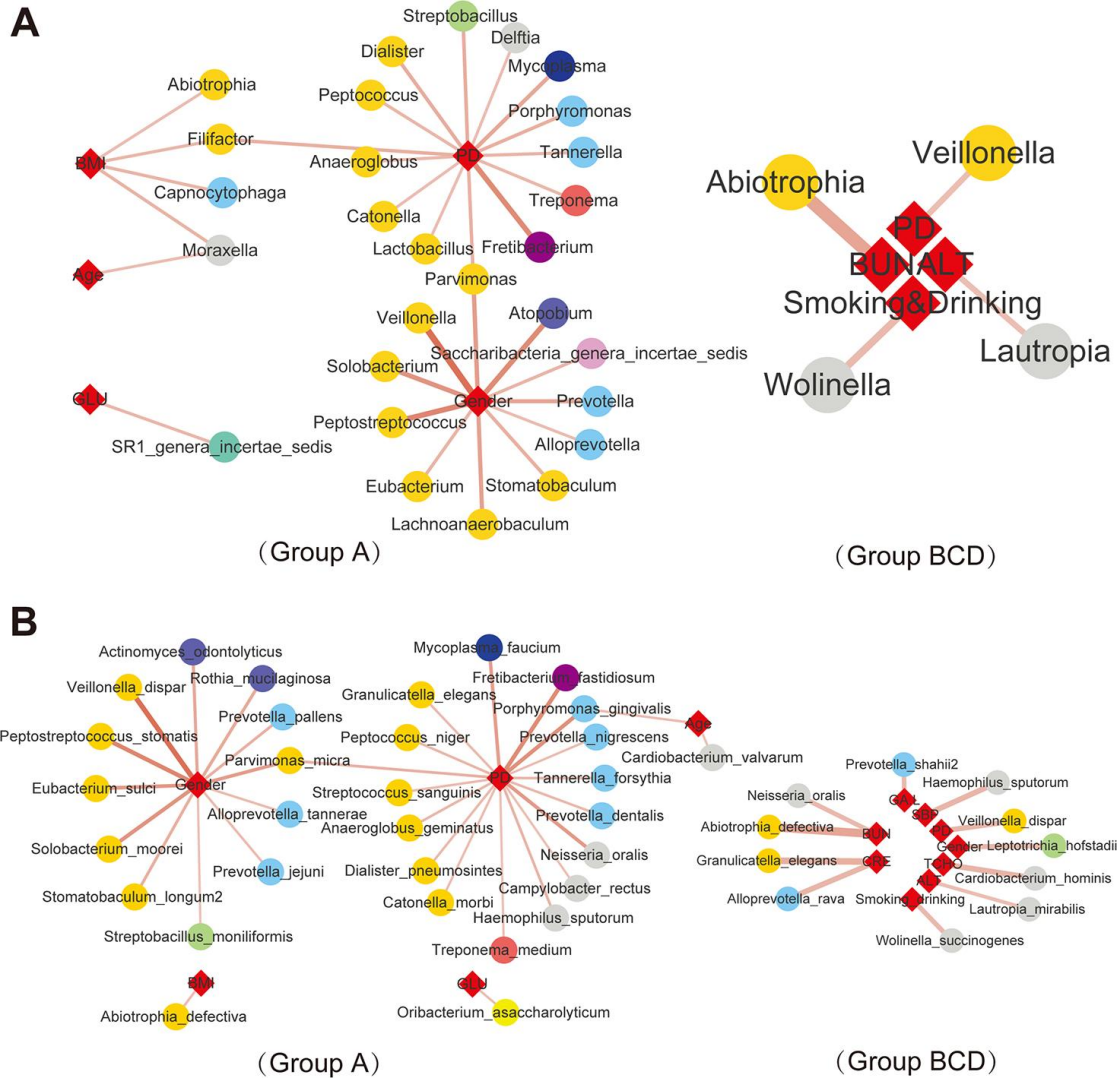
**Detection and testing of salivary microbial markers.** The four differential bacteria differential bacteria were applied for the succeeding random forest classification, and the ROC curve was used to evaluate the accuracy of sample classifications. The results showed that the accuracy rate of application of the differential bacteria as a biomarker to distinguish naïve diabetes patients from healthy people (**A**) and patients treated with metformin (**B**) or combined medication (**C**) could reach to 83.3%, 75%, and 75%, respectively.

As salivary microorganisms are closely related to diabetes indicators, salivary microbes could be used as potential biomarkers for the early warning of T2D. A random forest classifier model was applied for the group prediction, and a 5-fold cross-validation was used for the data preparation of the random forest model. The original data were randomly divided into 5 groups: one random subset was employed as the test set, and the remaining 4 groups were used as a training subset. Finally, five models were obtained, and the average accuracy of a model obtained from the test sets was used for the performance evaluation of the model. The random forest model directly produces the significance scores of a biomarker in the sample groupings, therefore it is conducive to finding the most important influencing factors. Differential bacterial species were evaluated by the random forest model, and the results showed that the order of importance for the four bacteria was *Selenomonas artemidis,* *Lactobacillus fermentum,* *Blautia wexlerae,* and *Nocardia coeliaca* (Supplementary Figure 13). To distinguish treatment-naïve diabetic patients from healthy nondiabetic people and those receiving insulin therapies and metformin treatment, the four differential bacteria were applied for the succeeding random forest classification, and the ROC curve was used to evaluate the accuracy of the sample classifications. The results showed that the accuracy rate of the application of the differential bacteria as biomarkers to distinguish treatment-naïve diabetic patients from nondiabetic people (Figure 5A) and patients treated with metformin (Figure 5B) or combined medication (Figure 5C) reached 83.3%, 75%, and 75%, respectively (Figure 5B).

Using the Random Forest model by *Lactobacillus_fermentum* and *Selenomonas_artemidis* established in this manuscript, we analyze the salivary microbiome data of two previous literatures for verification [27, 28], and the results show that the accuracy are 0.742 for distinguishing obese people with diabetes to those without diabetes [27]; and 0.746 for differentiating diabetes from non-diabetes [28], respectively (Supplementary Figure 14). Considering that two validation cohort was mainly from US population, these two salivary microbial biomarkers may have a global efficacy in assessing diabetes.

## DISCUSSION

Individuals with diabetes exhibit different patterns of gut microbiota compared to healthy individuals, and the gut microbiota of these individuals is considered to be in a dysbiosis state [29]. Saliva is considered to be the easiest available sample for disease risk prediction [30]. The change in saliva composition is closely related to the pathophysiological state of the body, and it is of great significance to explore disease-associated biomarkers in saliva [31]. Our study found that T2D could also significantly alter the salivary microbiota, while treatment did not lead to flora recovery. This finding is of great significance for the study of the pathogenesis and treatment mechanisms of diabetes and could be explained by two different mechanisms. First, elevated glucose levels in the saliva of diabetic patients could have impacted the oral environment, increasing the growth of certain bacteria at the expense of others [32, 33]; however, the change may be irreversible, and drug treatment cannot restore the oral environment to the original state. For example, elevated glucose in gingival crevicular fluid in diabetic patients may affect the growth of certain bacteria [34], and the bacterial composition in the subgingival plaque was altered [35]. The presence of *Porphyromonas gingivalis* in the periodontal pockets affects the glycemic index in diabetes [25], and the periodontitis-inducing cytokines TNF-α, IL-6 and IL-1β are also insulin antagonists [36], triggering irreversible changes in the body's immune response and inflammatory state. Second, hyperglycemia could have caused the acidification of the oral environment and perturbed the oral microbiota [37], the oral microbial diversity of diabetic patients decreases, and some bacteria have been "lost". These "lost" bacteria could not grow after the oral pH returned to normal after drug treatment. In addition, patients usually reduce the intake of starchy foods after they know their diabetes. Starchy foods probably affect the composition of the oral flora because only starch in the food can degrade in the mouth to produce glucose. However, a previous study found that regardless of how people with diabetes reduced their intake of starch, their oral glucose levels were always higher than normal [37], which eliminated the effects of starchy foods on the oral flora. T2DM is a state associated with a lack of diversity in the microbiota, and studies have reported that specific oral microbes are associated with diabetes [20, 25]. It has been observed that the microbiota is converted from healthy Gram-positive cocci and filaments to Gram-negative anaerobic bacteria in gingivitis. Qin et al. reported that the species enriched in the control samples included Clostridiales sp. SS3/4, *Eubacterium rectale*, *Faecalibacterium prausnitzii, Roseburia intestinalis* and *Roseburia inulinivorans* [8]. By contrast, T2D-enriched bacteria were *Bacteroides caccae*, *Clostridium hathewayi, Clostridium ramosum, Clostridium symbiosum, Eggerthella lenta* and *Escherichia coli*. However, these bacteria were not included in the differentiated oral bacteria in T2D in this study, suggesting that the oral microbiota and the intestinal flora play divergent roles when interacting with diabetes. In this study, the levels of *Faecalibacterium_prausnitzii* and *Lactobacillus spp.* were observed to be increased during the onset of diabetes but decreased after drug treatment, which was contrary to the previously reported results of the intestinal flora [38]. The reason may be that the oral cavity and the intestine are different microenvironments, leading to divergence in microbiota changes. Subsequent correlational analysis of the differential bacteria and clinical characteristics demonstrated that oral microbes were related to drug treatment and certain pathological changes, e.g., *Acinetobacter nosocomialis* was positively related to metformin, while *Haemophilus parainfluenzae* was positively associated with combined treatment. Abiotrophis was positively associated with blood urea nitrogen, Lautropia was positively associated with alanine aminotransferase, and Veillonella was positively associated with periodontitis. The correlation between salivary bacteria and clinical parameters might be related to disease occurrence and deserves in-depth study.

Periodontitis is an inflammatory disease induced by microbial infection that eventually destroys the connective tissue and bone supporting the teeth [39]; in addition, specific bacteria in the mouth are associated with the development and progression of periodontal disease [40]. Oral microbiota may be associated with diabetes, and there is evidence that diabetic patients are more likely to develop severe periodontal disease than healthy patients [41, 42]. At the same time, periodontitis makes blood sugar more difficult to control in T2DM patients [46], and periodontal disease could also worsen type 2 diabetes; thus, there is a two-way relationship between diabetes and periodontal disease [43, 44]. Patients with severe periodontal disease had higher levels of TNF-α in the blood circulation [45], and the cytokines TNF-α, IL-6 and IL-1β are insulin antagonists, which play an important role in the pathogenesis of periodontitis [36, 46]. Although there is some evidence that treatments aimed at reducing the burden of inflammation in periodontal disease can moderately improve glycemic control, a recent randomized trial of adults using nonsurgical periodontal treatment showed contradictory results [47]. In this study, the bacteria with significant differences in L, M, and H levels of periodontitis were not included in the aforementioned differential bacteria in the onset and treatment of diabetes, indicating that bacterial changes caused by periodontitis do not affect the bacterial differences caused by diabetes and treatment. Thus, this discovery diminishes the effect of periodontitis on the oral microbiota structure in an attempt to link the observed changes in the oral flora and the diabetic status with minimal impact from other factors.

It was previously found that the V3-V4 and V4-V5 regions of 16S rRNA yielded the most accurate results, regardless of sequencing technology and quality [48]. The capture of diversity using the primer pair spanning the V3-V4 hypervariable region had better capture when compared to the primer pair for the V1-V3 region [49]. Meanwhile, the V4 hypervariable region is traditionally selected for work as it provides adequate information for taxonomic classification of microbial communities and has demonstrated a lower error rate on the Illumina platform [50].

It is of great significance to explore disease-associated biomarkers in saliva [31], and metabolic markers have already been applied to the diagnosis, treatment and prognosis evaluation of periodontal disease and diabetes. Previous studies revealed that the level of α-2-macroglobulin in the saliva of T2D patients was significantly elevated, and its concentration was decreased after the control of blood glucose, suggesting that the detection of salivary α-2-macroglobulin level could be used to monitor the blood sugar in diabetic patients [51]. Studies also found that patients with T2D and periodontitis had decreased levels of melatonin in saliva, suggesting that melatonin in diabetes and teeth plays an important role in the pathogenesis of diseases and may become a key molecule in the diagnosis and therapy of these two diseases [52]. Barnes et al examined salivary metabolites in healthy and diabetic patients with or without periodontal disease and found that the levels of glucose and α-hydroxybutyrate in the saliva of diabetic patients increased, and the markers related to carbohydrates, lipids and oxidative stress changed significantly [53]. There are also studies that detected salivary blood glucose and glycated hemoglobin levels in patients with T2D and healthy people and found a positive correlation between them [54]. However, testing using saliva biomarkers is rare, and there is no report of diabetes detection and treatment effectiveness evaluation using saliva microbiota. In this study, we found significant differences between diabetic and nondiabetic individuals, and some differential bacteria emerged after treatment with metformin or combined medication. Our predictions with the random forest model had high accuracy, indicating that the use of saliva bacteria for disease diagnosis has great potential and is worthy of validation and optimization in the future.

## MATERIALS AND METHODS

### Ethics statement and samples collection

This study was reviewed and approved by the Ethical Committee of Shandong Academy of Medical Sciences. All volunteers gave their written informed consent prior to their inclusion of the study. All methods were performed in accordance with the relevant guidelines and regulations**.** Saliva specimens were obtained from Diabetes Research Hospital affiliated to Shandong Academy of Medical Sciences (Jinan city, Shandong province). Please refer to Supplementary Tables 1 and 2 for details of sampling patients.

Non-diabetic volunteers inclusion criteria: no antibiotics, glucocorticoids, immunosuppressive agents that stimulate the body's immune system have been used in the past 3 months; no probiotics have been used in large doses in the past 1 month; no local antibiotics treatment (such as that in mouth washes) have been used in the past 7 days; no infectious diseases such as hepatitis and tuberculosis; women not in pregnancy and lactation; without chronic diseases and clinical long-term medication, such as chronic functional diseases (such as lung, cardiovascular, gastrointestinal, liver and kidney, and tumors). Diabetes patient inclusion criteria: meet the WHO diagnostic criteria for type 2 diabetes; the other inclusion criteria are the same as those for non-diabetic volunteers. Exclusion criteria: those with malignant tumors; those with infectious diseases such as tuberculosis and viral hepatitis; those who take antibiotics or immunosuppressive agents for a long time; those with autoimmune diseases; those with blood diseases; those with obvious liver and kidney dysfunction; those with acute infection, trauma, or in other stressful state; those with a history of surgery; alcoholics (greater than 210g/week for male; greater than 140g/week for female); those with probiotics uptake; those with clinical trials of other drugs. Standards for mild to moderate periodontitis: each patients was examined and recorded his oral status includes oral hygiene, sacral stones and underarm stones, presence or absence of pigmentation on the tooth surface; and periodontal status includes gingival inflammation, periodontal pocket depth, gingival recession, and tooth looseness. The oral health status was then assessed by the dentist according to the condition of the examination record.

Saliva specimen collection: Volunteers did not eat after dinner on the day before the saliva sample collection, and brushed their teeth before going to bed; the volunteers did not brush their teeth on the day of collection, and saliva was collected 2h after breakfast (time is around 9:00). In detail, 15 min before sampling, the volunteers washed the oral cavity with sterile distilled water three times, each time the amount of sterile distilled water was 15ml. The volunteers collected saliva immediately after gargle, and the volunteers should not eat or drink, smoking or chewing gum in this 15 min. The patients should wash their hands and carry out other necessary cleaning measures before taking the sample; the collection container delivered to the patient should mark the donor's name and date; the subject keeps the saliva in the mouth for at least 1 min, and then collects the saliva sample using a microcentrifuge tube. In order to ensure that enough saliva is collected (2-3 mL, 2 specimens per person), this process usually needs to be repeated several times. Each sample was transferred to the laboratory within 20 minutes and immediately stored at -80 °C.

### DNA extraction, library construction and sequencing

### Extraction of DNA

Total DNA was extracted with CTAB/SDS method. 1% agarose gels was used for checking the DNA concentration and purity. DNA was diluted to 1ng/μl using sterile water based on the obtained concentration.

### Library construction and sequencing

Genome DNA from all the samples was used as amplification templates. PCR primers were from the V3-V4 region of 16S rDNA, specific primers used was: forward primer, 5'-ACTCCTACGGGAGGCAGCA-3'; and reverse primer, 5'-GGACTACHVGGGTWTCTAAT-3'. All PCR reactions were carried out in 30μL reactions with 15μL of Phusion® High-Fidelity PCR Master Mix (New England Biolabs); 0.2μM of forward and reverse primers, and about 10 ng templates DNA. Thermal cycling included a denaturation step at 95°C for 5 min, followed by 30 cycles of denaturation at 95°C for 30s, annealing at 50°C for 30s, and elongation at 72°C for 40s. Finally 72°C for 7 min.

Then the PCR products were purified with GeneJET Gel Extraction Kit (Thermo Scientific) and qualified by electrophoresis on 2% agarose gel, samples with single amplification product were chosen for further experiments. The library was sequenced on an Illumina Hiseq 2500 platform at Novogene company (Beijing, China).

### Data processing

In data preprocessing, quality filtering and analysis of the Raw sequence were performed using the next-generation microbiome bioinformatics platform (QIIME2 version 2018.6 pipeline). The quality filtering algorithm, a software package included in Usearch, was used to identify exact sequence variants (ESVs). Alpha and beta-diversity analyses were performed in R using the phyloseq package. Alpha diversity was calculated by Faith's Phylogenetic Diversity, Shannon index, and observed OTUs. Principal coordinate analysis (PCoA) was analyzed based on unweighted UniFrac distances, a method for computing differences between microbial communities based on phylogenetic information. Permutational multivariate analysis of variance (PERMANOVA, R function adonis (vegan, 999 permutations)) was employed to analyze statistical differences in beta diversity. Bray-Curtis dissimilarity is an indicator used to measure differences in taxonomic composition in ecology, and Jaccard Distance is to measure the difference between two groups. Benjamini–Hochberg false discovery rate (fdr) correction was applied to correct multiple hypothesis testing. The contribution of periodontal disease to the weighted, unweighted, UniFrac, bray Curtis, and jaccard dissimilarities was also evaluated using PERMANOVA (R function adonis (vegan), 999 permutations), and the dissimilarity matrix was decomposed into “variance”. We used Usearch to cluster the Effective Tags of all samples, cluster the sequences into 1055 Operational Taxonomic Units (OTUs) with 97% identity, and then perform species annotation on the representative sequences of OTUs according to the database Ribosomal Database Project (The database version is RDP Release 11.5). At the genus and species levels, differences were analyzed between the A, B, C, and D groups. The average relative abundance of less than 0.02%, and the frequency of occurrence in the sample less than 30% (R package: "dplyr") was screened out for the Wilcoxon test. The species with p <= 0.05 were selected with R package: "pheatmap" for the heat map, and the average relative abundance in the group was demonstrated with a histogram (R package: "ggplot2"). Correlation analysis was performed at the genus and species levels, all the differential genera and species identified in the variation analysis were pooled for spearman correlation analysis of their correlation with all the bacteria and clinical parameters after 0.002% and 30% screening (R package: "Hmisc"). The genera and Species with padj<=0.05, |cor|>=0.5 were shown with a network map (software cytoscape, R package “pheatmap”). To further explore the relationship between salivary microbiota and the degree of diabetes, we used a random forest algorithm to predict the classification of samples. Random forest regression was done with 1,000 regression trees based on 5-fold cross-validation and the Random Forest regressor in the R programming environment. A random drawn 80% of samples were used for model training and the remaining 20% were used for validation. The predicted results were shown with the roc curve (R package: "proc"). In the random forest prediction classification algorithm, the contribution of different species can be kown. The top four species were selected and the random forest algorithm prediction classification performed.

## Supplementary Material

Supplementary Figures

Supplementary Tables 1, 8, 9

Supplementary Table 2

Supplementary Table 3

Supplementary Table 4

Supplementary Table 5

Supplementary Table 6

Supplementary Table 7
