# Supplementary material for: Changes of saliva microbiota in the onset and after the treatment of diabetes in patients with periodontitis
Source: Aging (Albany NY). 2020 Jul 7;12(13):13090–114. doi: 10.18632/aging.103399 (PMC7377876; doi:10.18632/aging.103399)
Supplement: Supplementary Figures [file aging-12-103399-s008..pdf]

SUPPLEMENTARY FIGURES

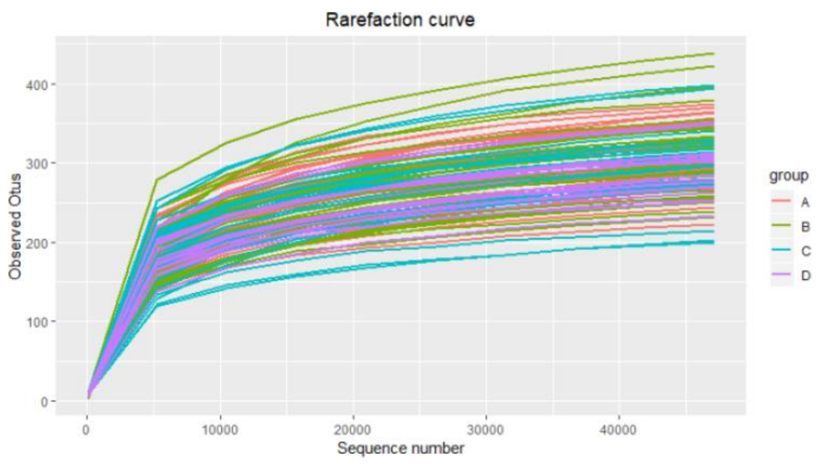

Supplementary Figure 1. Rarefaction curve: the abscissa is the number of randomly selected sequences and the ordinate is the number of OTUs clustered based on the number of sequences. Each curve represents a sample, and the curve tends to be stable, indicating that the number of extracted sequences is enough to reflect the OTUs of the sample.

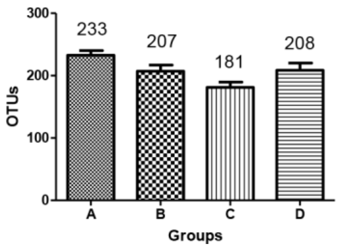

Supplementary Figure 2. The number of OTUs in each group.

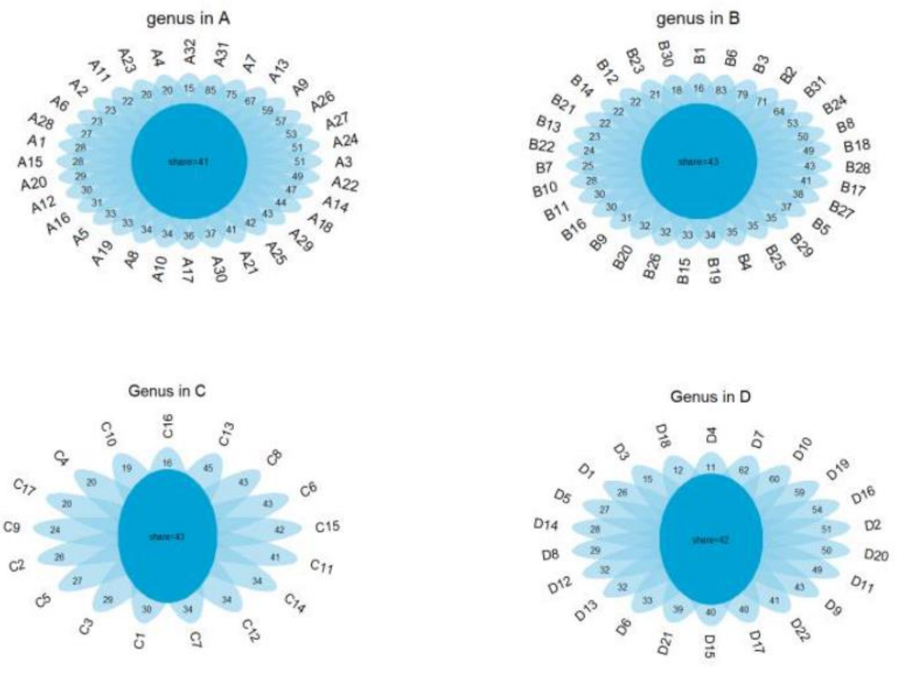

Supplementary Figure 3. The "core microbiome" at the genus level.

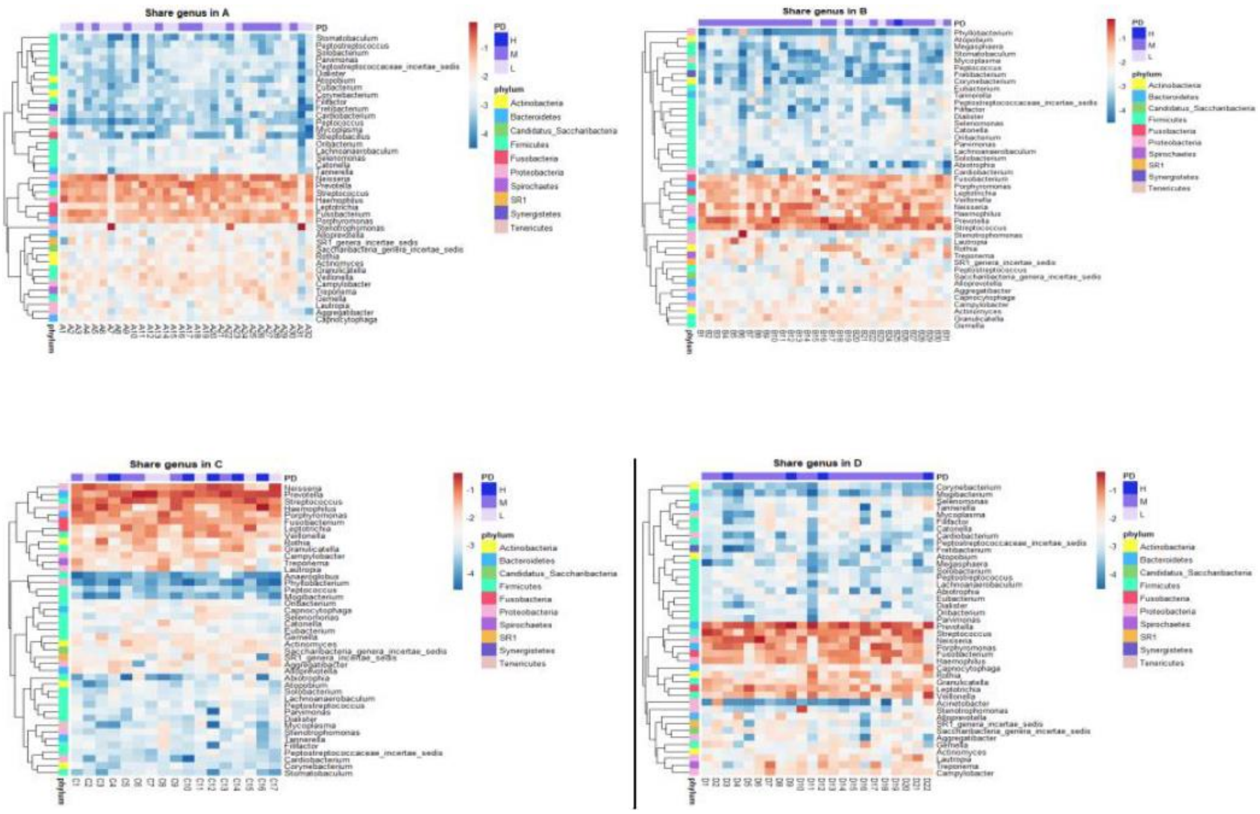

Supplementary Figure 4. The abundances of salivary "core microbiome" among individuals at the genus level.

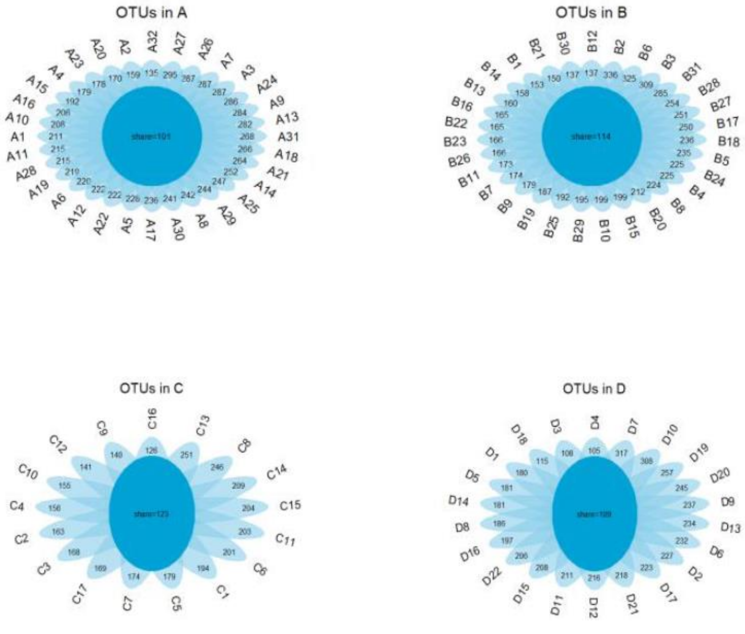

Supplementary Figure 5. The salivary "core microbiome" at the OTU level.

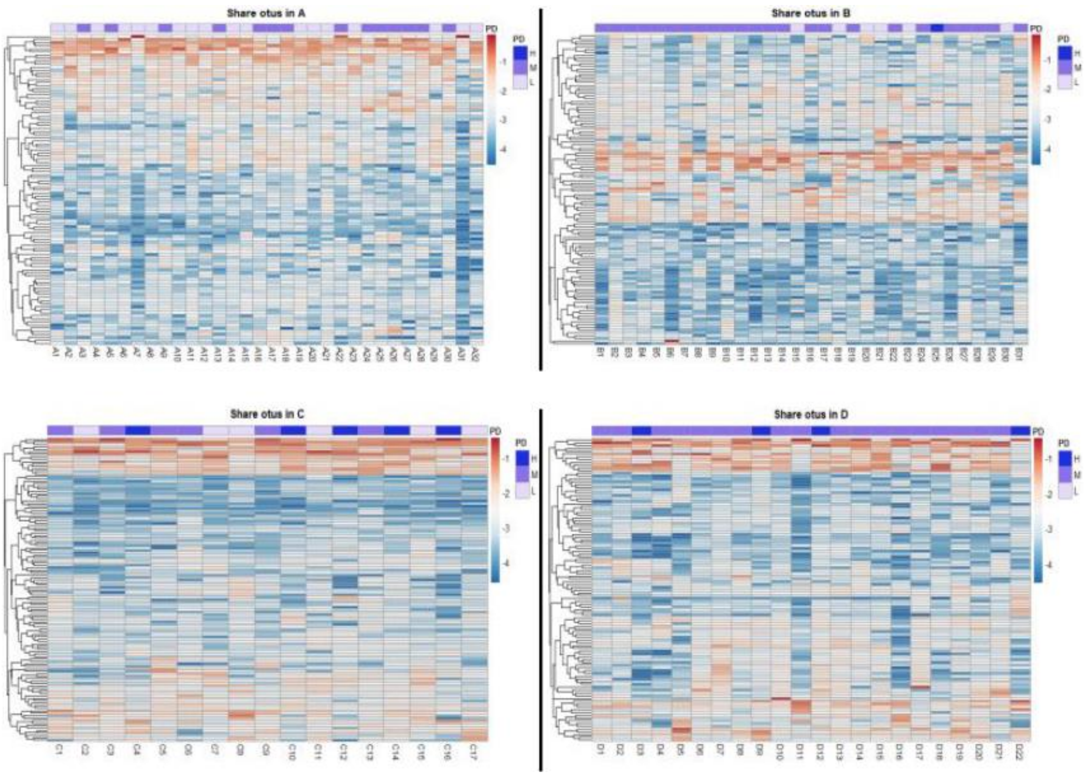

Supplementary Figure 6. The abundance of salivary "core microbiome" among individuals at the OTU level.

Bray Curtis

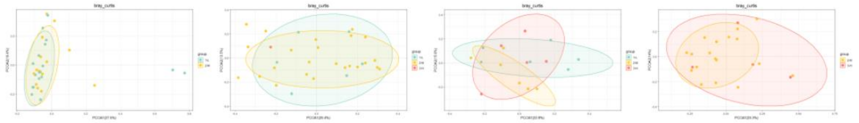

Adonis p value: (A)p=0.28 (B) p=0.43 (C) p=0.26 (D) p=0.82

Jaccard

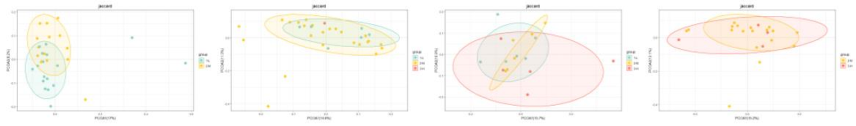

Adonis p value: (A) p=0. 40 (B) p=0.82 (C) p=0.54 (D) p=0.90

Unweighted Unifrac

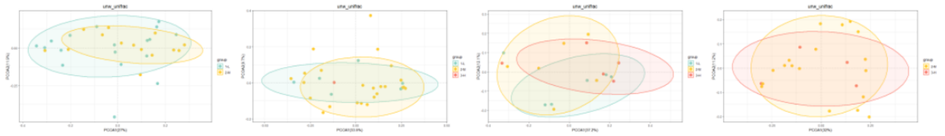

Adonis p value: (A) p=0.23 (B) p=0.73 (C) p=0.51 (D) p=0.76

Weighted Unifrac

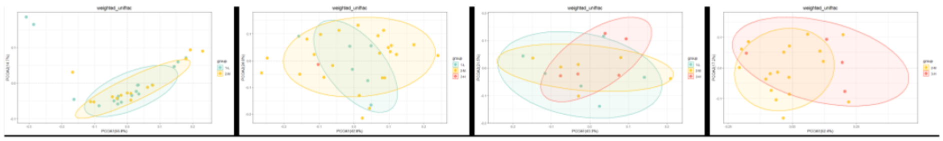

Adonis p value: (A) p=0.40 (B) p=0.65 (C) p=0.69 (D) p=0.22

Supplementary Figure 7. Beta diversity of periodontitis associated bacteria in each group.

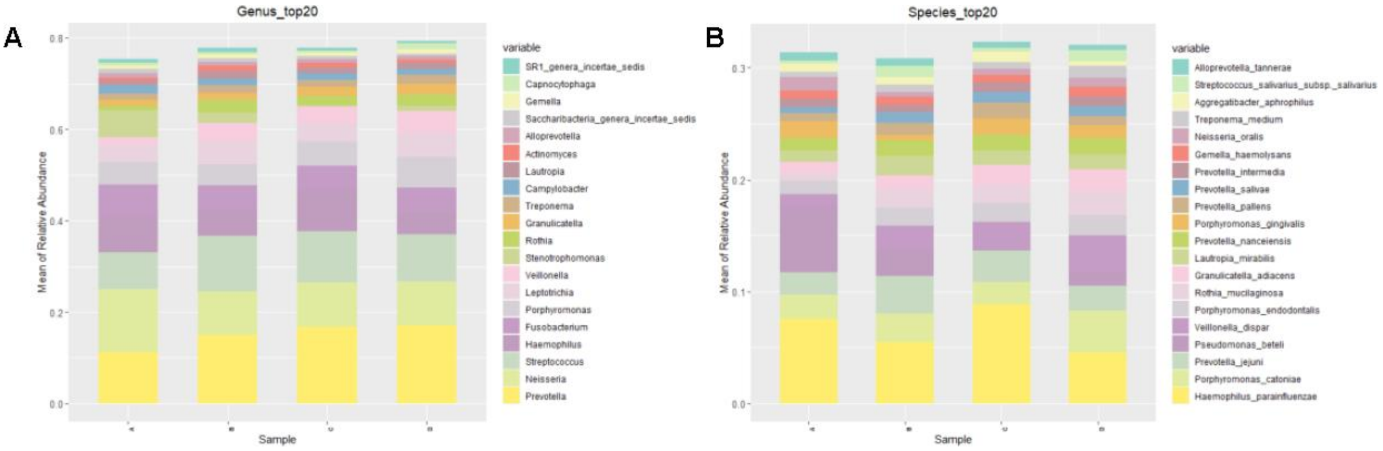

Supplementary Figure 8. The top 20 abundant genus (**A**) and species (**B**) of salivary microbiome in the non-diabetic control (Group A), diabetes (Group B), metformin (Group C) and combination treatment (Group D) of diabetes.

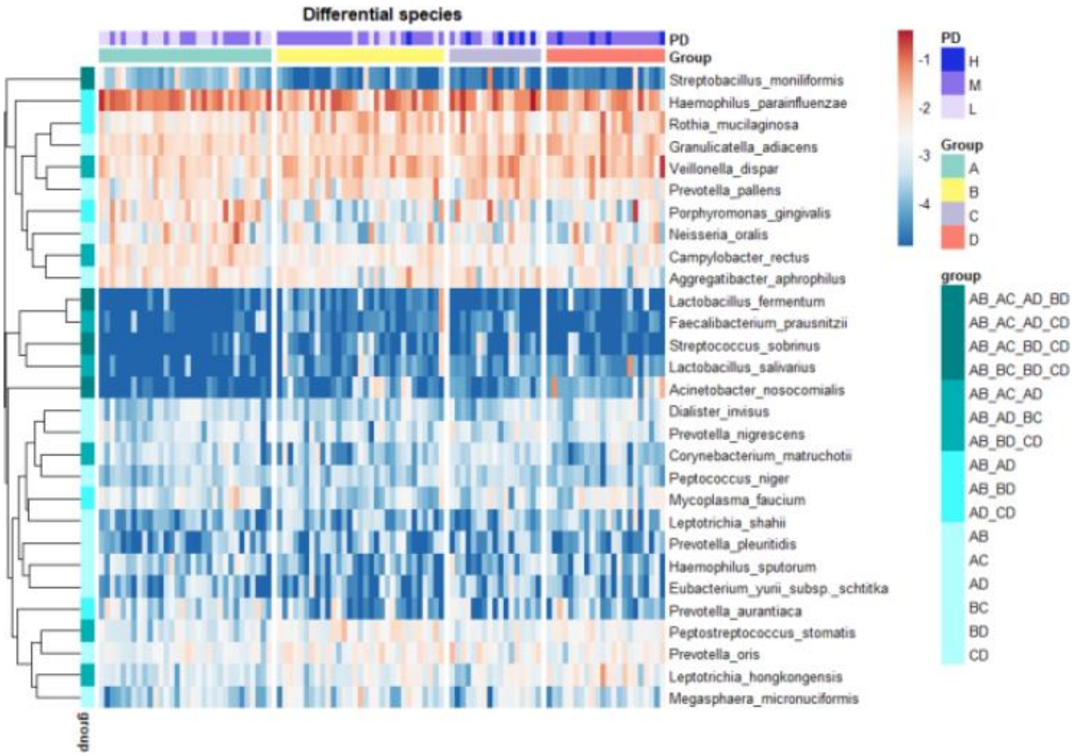

Supplementary Figure 9. The differential species of salivary microbiome in the non-diabetic control (Group A), diabetes (Group B), metformin (Group C) and combination treatment (Group D) of diabetes.

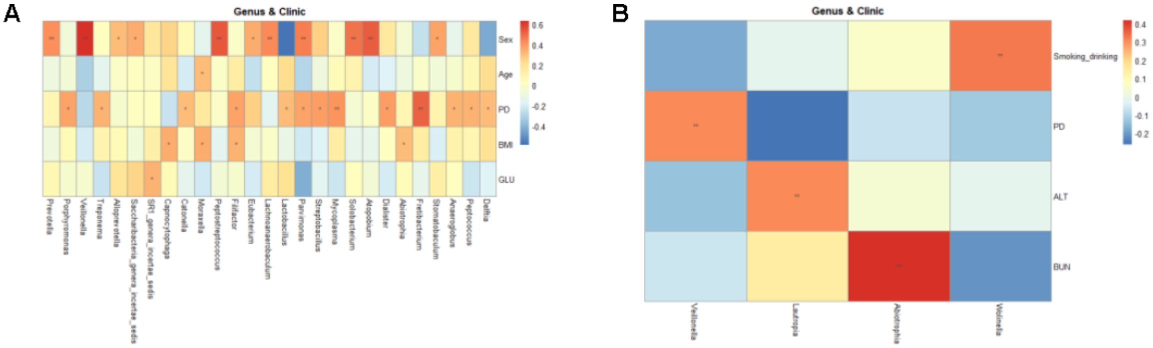

Supplementary Figure 10. Correlation between differential bacteria in the microbiota and various clinical parameters in each group at the genus level. (**A**) Group A; (**B**) Group B, C and D.

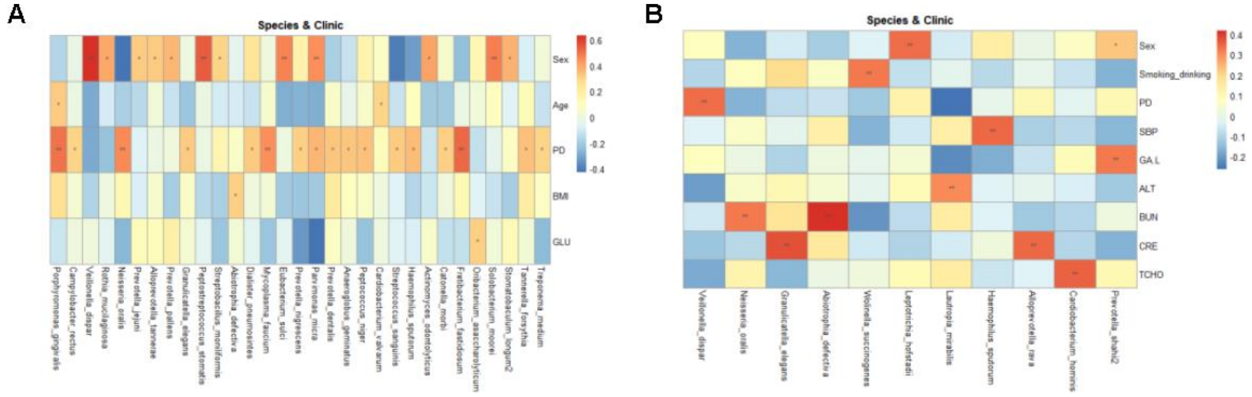

Supplementary Figure 11. Correlation between differential bacteria in the microbiota and various clinical parameters in each group at the species level. (**A**) Group A; (**B**) Group B, C and D.

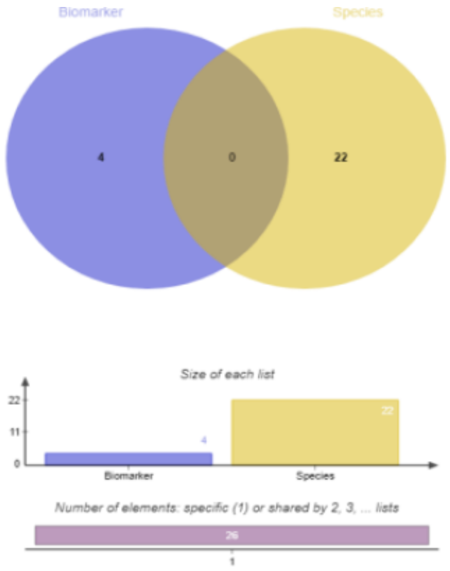

Supplementary Figure 12. The intersection of biomarkers, *Blautia_wexlerae, Lactobacillus_fermentum, Nocardia_coeliaca and Selenomonas_artemidis*, with the bacteria that have larger correlation with OTUs.

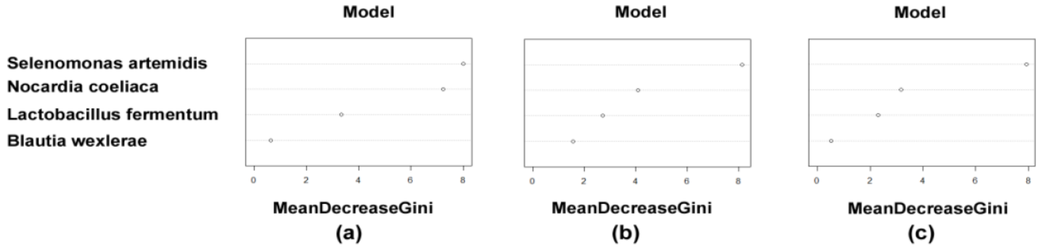

Supplementary Figure 13. The differential bacteria species were evaluated by the random forest model.

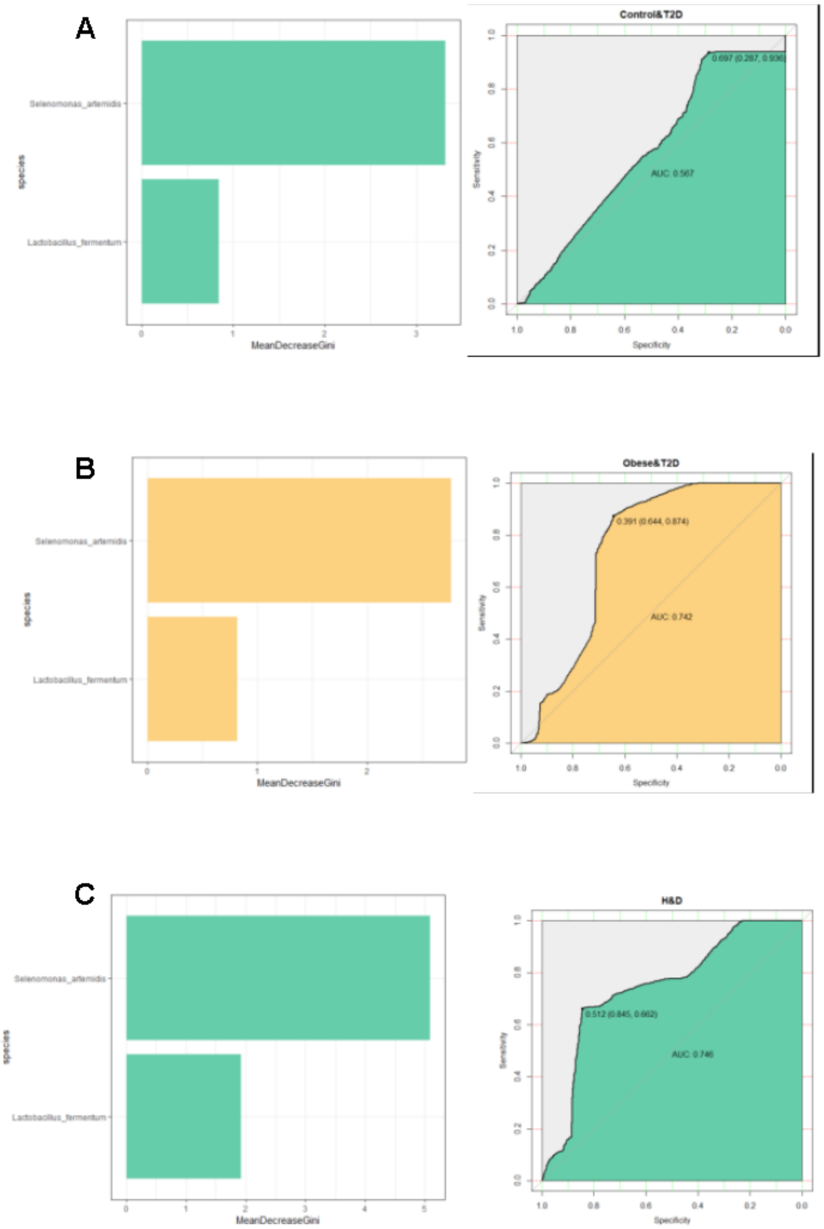

Supplementary Figure 14. Using the Random Forest model by *Lactobacillus_fermentum* and *Selenomonas_artemidis* to analyze the data of two previous literatures [Janem et al., 2017]. (**A** and **B**); [Sabharwal et al., 2019] (**C**).
