# Supplementary material for: Changes of saliva microbiota in the onset and after the treatment of diabetes in patients with periodontitis
Source: Aging (Albany NY). 2020 Jul 7;12(13):13090–114. doi: 10.18632/aging.103399 (PMC7377876; doi:10.18632/aging.103399)
Supplement: Supplementary Tables 1, 8, 9 [file aging-12-103399-s007..pdf]

SUPPLEMENTARY TABLES

Supplementary Table 1. The information of the four groups of people.

Groups	A	B	C	D
Number of people	32	31	17	22
Sex	16M, 16F	23M, 8F	13M, 4F	14M, 8F
Symptoms	PD	PD+Diabetes	PD+Diabetes	PD+Diabetes
Treatments	Non	Non	Metformin	Insulin+metformin or other hypoglycemic drugs
PD	18L, 14M	6L, 24M, 1H	6L, 6M, 5H	18M, 4H
BMI	24.77±2.70	26.54±5.40	27.14±4.41	26.36±3.46
Glu	5.38±0.48	12.43±4.66	10.54±3.51	11.67±3.22
ALT	20.91±7.69	37.55±31.99	30.43±18.09	24.67±8.32
AST	17.36±2.82	24.24±16.49	22.85±11.70	18.25±3.81
BUN	4.79±1.37	5.379±1.23	5.03±1.10	5.71±1.26
CRE	62.43±9.70	64.34±10.66	68.65±11.45	56.96±10.16
UA	319.79±102.03	337.26±126.36	330.14±92.26	286.46±86.61
TG	1.90±1.71	2.69±2.55	1.85±0.80	1.89±1.55
TCHO	5.02±0.44	5.80±1.11	5.06±0.78	5.29±0.81
SBP	No data	134.87±15.10	137.35±12.38	143.41±15.77
GA.L	No data	26.99±9.28	27.31±10.33	28.37±4.69
HDL-C	1.34±0.27	1.27±0.38	1.27±0.30	1.39±0.41

Their Fasting blood sugar (Glu), Body Mass Index (BMI), periodontitis (PD), blood urea nitrogen (BUN), Systolic blood pressure (SBP), Total cholesterol (TCHO), Glycosylated serum protein (GA.L), Creatinine (Cre) and alanine aminotransferase (ALT) were measured.

Please browse Full Text version to see the data of Supplementary Tables 2 to 7.

Supplementary Table 2. Details of the sampled volunteers.

Supplementary Table 3. Assessment of the data quality.

Supplementary Table 4. Annotated microbiota to different taxa.

Supplementary Table 5. The periodontitis-associated taxa in each group.

Supplementary Table 6. Taxa with significant difference in content between groups

Supplementary Table 7. Genera and species with significant difference in content between groups.

Supplementary Table 8. ADONIS analysis of the contribution of age, sex and OTUs to sample differences.

R^2, P	Age	Sex	OTUs of each sample
Bray_curtis	0.00896, 0.545	0.01229, 0.227	0.0678, 0.001
Jaccard	0.01147, 0.181	0.01165, 0.137	0.07751, 0.001
Unweighted_unifrac	0.01112, 0.275	0.00877, 0.495	0.12732, 0.001
Weighted_unifrac	0.01112, 0.276	0.00877, 0.503	0.12732, 0.001

Supplementary Table 9. Correlation analysis between the microbiota and OTUs, 22 species were obtained when p <= 0.05, the absolute value of the correlation coefficient |cor|> = 0.3.

Species had p<= 0.05 and |cor|>= 0.3
Pseudomonas_beteli
Porphyromonas_gingivalis
Campylobacter_rectus
Neisseria_oralis
Streptobacillus_moniliformis
Phyllobacterium_myrsinacearum
Dialister_pneumosintes
Mycoplasma_faucium
Prevotella_denticola
Wolinella_succinogenes
Dialister_invisus
Prevotella_dentalis
Anaeroglobus_geminatus
Selenomonas_noxia
Lautropia_mirabilis
Leptotrichia_shahii
Fretibacterium_fastidiosum
Selenomonas_infelix
Selenomonas_sputigena
Tannerella_forsythia
Treponema_medium
Treponema_amylovorum
