# Supplementary material for: Changes of saliva microbiota in the onset and after the treatment of diabetes in patients with periodontitis
Source: Aging (Albany NY). 2020 Jul 7;12(13):13090–114. doi: 10.18632/aging.103399 (PMC7377876; doi:10.18632/aging.103399)
Supplement: Supplementary Table 2 [file aging-12-103399-s003..docx]

**Supplementary Table 2. Details of the sampled volunteers.**

| **Samples** | **Sex** | **Age** | **PD** | **Diabetes (years)** |
| --- | --- | --- | --- | --- |
| A1 | F | 38 | PD(L) | 0 |
| A10 | M | 51 | PD(L) | 0 |
| A11 | F | 50 | PD(L) | 0 |
| A12 | M | 42 | PD(L) | 0 |
| A13 | M | 48 | PD(M) | 0 |
| A14 | M | 42 | PD(L) | 0 |
| A15 | F | 52 | PD(L) | 0 |
| A16 | F | 45 | PD(M) | 0 |
| A17 | M | 45 | PD(M) | 0 |
| A18 | M | 44 | PD(M) | 0 |
| A19 | F | 48 | PD(L) | 0 |
| A2 | F | 40 | PD(L) | 0 |
| A20 | F | 46 | PD(L) | 0 |
| A21 | F | 42 | PD(L) | 0 |
| A22 | M | 42 | PD(M) | 0 |
| A23 | F | 40 | PD(L) | 0 |
| A24 | M | 48 | PD(M) | 0 |
| A25 | F | 40 | PD(M) | 0 |
| A26 | M | 69 | PD(M) | 0 |
| A27 | M | 62 | PD(M) | 0 |
| A28 | M | 63 | PD(M) | 0 |
| A29 | F | 63 | PD(L) | 0 |
| A3 | M | 41 | PD(M) | 0 |
| A30 | F | 48 | PD(M) | 0 |
| A31 | M | 48 | PD(L) | 0 |
| A32 | F | 47 | PD(L) | 0 |
| A4 | F | 45 | PD(L) | 0 |
| A5 | F | 49 | PD(M) | 0 |
| A6 | M | 52 | PD(L) | 0 |
| A7 | M | 55 | PD(L) | 0 |
| A8 | M | 56 | PD(L) | 0 |
| A9 | F | 73 | PD(M) | 0 |
| B1 | M | 33 | PD(M) | <1 |
| B10 | M | 45 | PD(M) | 2 |
| B11 | M | 69 | PD(M) | <1 |
| B12 | M | 30 | PD(M) | 4 |
| B14 | M | 72 | PD(M) | 1 |
| B15 | F | 71 | PD(M) | 6 |
| B16 | F | 41 | PD(L) | 6 |
| B17 | M | 53 | PD(M) | <1 |
| B18 | M | 54 | PD(M) | <1 |
| B19 | M | 48 | PD(L) | 5 |
| B2 | M | 48 | PD(M) | 16 |
| B20 | F | 75 | PD(M) | 20 |
| B21 | M | 44 | PD(L) | <1 |
| B22 | M | 58 | PD(L) | 2 |
| B23 | F | 63 | PD(M) | <1 |
| B24 | M | 37 | PD(L) | <1 |
| B25 | M | 56 | PD(M) | 5 |
| B26 | M | 76 | PD(H) | 1 |
| B27 | M | 48 | PD(M) | 1 |
| B28 | F | 64 | PD(M) | 2 |
| B29 | M | 44 | PD(M) | 3 |
| B3 | M | 34 | PD(M) | 2 |
| B30 | M | 56 | PD(M) | 1 |
| B31 | M | 56 | PD(L) | 1 |
| B32 | M | 47 | PD(M) | 8 |
| B4 | F | 44 | PD(M) | <1 |
| B5 | M | 51 | PD(M) | <1 |
| B6 | M | 49 | PD(M) | 3 |
| B7 | F | 62 | PD(M) | 18 |
| B8 | F | 25 | PD(M) | <1 |
| B9 | M | 64 | PD(M) | 1 |
| C1 | F | 52 | PD(M) | 18 |
| C10 | M | 64 | PD(M) | 10 |
| C11 | M | 60 | PD(M) | 19 |
| C12 | M | 57 | PD(H) | 13 |
| C13 | M | 55 | PD(M) | 10 |
| C14 | F | 53 | PD(M) | 20 |
| C15 | M | 43 | PD(M) | 3 |
| C16 | M | 50 | PD(M) | 7 |
| C17 | F | 69 | PD(M) | 18 |
| C18 | M | 60 | PD(M) | 10 |
| C19 | F | 65 | PD(M) | 20 |
| C2 | F | 50 | PD(M) | 7 |
| C20 | M | 68 | PD(M) | 15 |
| C21 | F | 47 | PD(M) | 18 |
| C22 | F | 71 | PD(H) | 11 |
| C3 | M | 73 | PD(H) | 26 |
| C4 | M | 57 | PD(M) | 13 |
| C5 | M | 54 | PD(M) | 14 |
| C6 | M | 64 | PD(M) | 12 |
| C7 | M | 54 | PD(M) | 10 |
| C8 | F | 65 | PD(M) | 7 |
| C9 | M | 37 | PD(H) | 31 |
| D1 | M | 56 | PD(M) | 6 |
| D10 | M | 48 | PD(H) | 14 |
| D11 | M | 47 | PD(L) | 2 |
| D12 | F | 53 | PD(H) | 15天 |
| D13 | M | 68 | PD(M) | 3 |
| D14 | M | 76 | PD(H) | 1 |
| D15 | M | 65 | PD(L) | 4 |
| D16 | M | 55 | PD(H) | 14 |
| D17 | M | 58 | PD(L) | <1 |
| D2 | M | 31 | PD(L) | 2 |
| D3 | F | 68 | PD(M) | 15 |
| D4 | M | 75 | PD(H) | 4 |
| D5 | F | 58 | PD(M) | 15 |
| D6 | M | 44 | PD(M) | <1 |
| D7 | M | 49 | PD(L) | 5 |
| D8 | M | 33 | PD(L) | 1 |
| D9 | F | 55 | PD(M) | 6 |
