# Supplementary material for: Changes of saliva microbiota in the onset and after the treatment of diabetes in patients with periodontitis
Source: Aging (Albany NY). 2020 Jul 7;12(13):13090–114. doi: 10.18632/aging.103399 (PMC7377876; doi:10.18632/aging.103399)
Supplement: Supplementary Table 3 [file aging-12-103399-s004..docx]

**Supplementary Table 3. Assessment of the data quality.**

| Sample Name | Raw Reads(#) | Clean Reads(#) | Base(nt) | AvgLen  (nt) | Q20 | GC% | Effective% |
| --- | --- | --- | --- | --- | --- | --- | --- |
| A1 | 87377 | 80139 | 33852932 | 422 | 88.51 | 51.92 | 91.72 |
| A2 | 84445 | 80180 | 33755136 | 420 | 88.15 | 51.83 | 94.95 |
| A3 | 84082 | 80231 | 33554928 | 418 | 88.78 | 51.28 | 95.42 |
| A4 | 93028 | 87605 | 36735014 | 419 | 88.33 | 52.02 | 94.17 |
| A5 | 87840 | 80032 | 33652259 | 420 | 89.04 | 51.38 | 91.11 |
| A6 | 96528 | 89108 | 36965169 | 414 | 87.22 | 51.4 | 92.31 |
| A7 | 83790 | 80021 | 33482967 | 418 | 88.1 | 51.11 | 95.5 |
| A8 | 86826 | 80244 | 34042241 | 424 | 89.01 | 52.89 | 92.42 |
| A9 | 86136 | 80109 | 33697192 | 420 | 88.51 | 51.41 | 93 |
| A10 | 86528 | 80072 | 33398107 | 417 | 88.96 | 50.14 | 92.54 |
| A11 | 82001 | 80128 | 33551918 | 418 | 87.93 | 51.4 | 97.72 |
| A12 | 79452 | 77233 | 32181606 | 416 | 87.72 | 50.86 | 97.21 |
| A13 | 88123 | 80088 | 33738287 | 421 | 87.44 | 51.81 | 90.88 |
| A14 | 86325 | 80216 | 33612040 | 419 | 87.95 | 51.27 | 92.92 |
| A15 | 88508 | 80223 | 34034109 | 424 | 87.13 | 52.85 | 90.64 |
| A16 | 83115 | 80177 | 33831291 | 421 | 88.75 | 51.71 | 96.47 |
| A17 | 82306 | 80312 | 33740493 | 420 | 88.87 | 51.31 | 97.58 |
| A18 | 84971 | 80167 | 33792919 | 421 | 90.13 | 52.22 | 94.35 |
| A19 | 86189 | 80274 | 33494260 | 417 | 88.73 | 51.28 | 93.14 |
| A20 | 85599 | 80159 | 33455884 | 417 | 88.93 | 52.17 | 93.64 |
| A21 | 87552 | 80156 | 33587508 | 419 | 88.15 | 51.44 | 91.55 |
| A22 | 85482 | 80239 | 33744166 | 420 | 88.48 | 51.41 | 93.87 |
| A23 | 83696 | 79581 | 33122569 | 416 | 86.35 | 50.8 | 95.08 |
| A24 | 74957 | 72404 | 30092716 | 415 | 87.65 | 50.77 | 96.59 |
| A25 | 86504 | 80063 | 33587485 | 419 | 88.29 | 51.62 | 92.55 |
| A26 | 86953 | 80316 | 33530277 | 417 | 88.27 | 51.26 | 92.37 |
| A27 | 83293 | 80122 | 33738398 | 421 | 88.1 | 51.61 | 96.19 |
| A28 | 81909 | 80241 | 33435049 | 416 | 88.22 | 51.4 | 97.96 |
| A29 | 79055 | 74005 | 31224577 | 421 | 87.72 | 52.04 | 93.61 |
| A30 | 86950 | 81645 | 33964865 | 416 | 86.32 | 50.64 | 93.9 |
| A31 | 84262 | 80219 | 33603912 | 418 | 87.8 | 51.43 | 95.2 |
| A32 | 85945 | 80255 | 33510741 | 417 | 86.85 | 51.6 | 93.38 |
| B1 | 88939 | 80062 | 33763682 | 421 | 88.06 | 51.75 | 90.02 |
| B2 | 84008 | 80113 | 33533278 | 418 | 88.24 | 51.33 | 95.36 |
| B3 | 105738 | 95359 | 39910875 | 418 | 84.32 | 51.16 | 90.18 |
| B4 | 88669 | 80239 | 33829571 | 421 | 87.85 | 51.86 | 90.49 |
| B5 | 74688 | 70437 | 29412363 | 417 | 87.6 | 51.37 | 94.31 |
| B6 | 84387 | 80238 | 33608038 | 418 | 87.35 | 51.99 | 95.08 |
| B7 | 83346 | 78749 | 32978617 | 418 | 87.58 | 51.82 | 94.48 |
| B8 | 82825 | 80196 | 33401100 | 416 | 87.35 | 49.82 | 96.83 |
| B9 | 85267 | 83206 | 34668098 | 416 | 88.2 | 51.63 | 97.58 |
| B10 | 72038 | 67518 | 28170892 | 417 | 87.3 | 51.68 | 93.73 |
| B11 | 87897 | 80057 | 33497691 | 418 | 86.95 | 51.22 | 91.08 |
| B12 | 83704 | 80248 | 33676520 | 419 | 87.68 | 51.63 | 95.87 |
| B14 | 77175 | 75469 | 31482473 | 417 | 87.92 | 51.81 | 97.79 |
| B15 | 82177 | 80088 | 33850090 | 422 | 85.88 | 52.62 | 97.46 |
| B16 | 82394 | 76187 | 31977664 | 419 | 87.7 | 51.42 | 92.47 |
| B17 | 71376 | 67743 | 28216914 | 416 | 87.69 | 52.08 | 94.91 |
| B18 | 84226 | 80239 | 33808324 | 421 | 87.75 | 51.57 | 95.27 |
| B19 | 81487 | 75588 | 31587502 | 417 | 87.42 | 51.13 | 92.76 |
| B20 | 92001 | 86319 | 36346545 | 421 | 88.03 | 51.57 | 93.82 |
| B21 | 82905 | 80063 | 33529812 | 418 | 87.92 | 51.24 | 96.57 |
| B22 | 88372 | 80134 | 33724371 | 420 | 87.65 | 51.93 | 90.68 |
| B23 | 72888 | 71060 | 29305221 | 412 | 86.34 | 50.86 | 97.49 |
| B24 | 61265 | 57391 | 23964491 | 417 | 87.94 | 52.51 | 93.68 |
| B25 | 84654 | 81483 | 34178727 | 419 | 88.2 | 51.52 | 96.25 |
| B26 | 81070 | 78904 | 32682393 | 414 | 87.87 | 51.21 | 97.33 |
| B27 | 87349 | 80238 | 33609654 | 418 | 87.61 | 51.45 | 91.86 |
| B28 | 61717 | 56412 | 23592531 | 418 | 87.57 | 51.67 | 91.4 |
| B29 | 86095 | 80200 | 33723554 | 420 | 87.25 | 51.17 | 93.15 |
| B30 | 88412 | 80061 | 33557180 | 419 | 88.2 | 51.29 | 90.55 |
| B31 | 83822 | 80197 | 33579187 | 418 | 88.96 | 51.98 | 95.68 |
| B32 | 86462 | 80136 | 33470216 | 417 | 88.57 | 51.44 | 92.68 |
| C1 | 86601 | 80280 | 33786932 | 420 | 88.42 | 51.71 | 92.7 |
| C2 | 100838 | 94285 | 39473868 | 418 | 89.21 | 51.07 | 93.5 |
| C3 | 84922 | 80038 | 33435753 | 417 | 89.01 | 51.77 | 94.25 |
| C4 | 79367 | 75396 | 31437294 | 416 | 87.77 | 51.76 | 95 |
| C5 | 86444 | 80252 | 33739657 | 420 | 88.91 | 51.38 | 92.84 |
| C6 | 86097 | 80129 | 33620508 | 419 | 88.48 | 51.45 | 93.07 |
| C7 | 81930 | 80118 | 33345054 | 416 | 88.74 | 51.06 | 97.79 |
| C8 | 86327 | 80124 | 33888208 | 422 | 88.49 | 51.48 | 92.81 |
| C9 | 88123 | 80083 | 33788852 | 421 | 89.31 | 51.33 | 90.88 |
| C10 | 84536 | 80274 | 33732411 | 420 | 88.72 | 51.71 | 94.96 |
| C11 | 84024 | 80013 | 33700815 | 421 | 88.07 | 52.59 | 95.23 |
| C12 | 74754 | 72552 | 30533021 | 420 | 87.3 | 51.35 | 97.05 |
| C13 | 80247 | 76617 | 32087485 | 418 | 87.69 | 51.47 | 95.48 |
| C14 | 85693 | 80227 | 33456997 | 417 | 87.62 | 51.08 | 93.62 |
| C15 | 82980 | 80355 | 33588465 | 418 | 86.47 | 51.38 | 96.84 |
| C16 | 86573 | 80108 | 33334219 | 416 | 89.16 | 50.46 | 92.53 |
| C17 | 63587 | 59201 | 24864705 | 420 | 86.76 | 51.59 | 93.1 |
| D1 | 86070 | 80101 | 33781097 | 421 | 89.41 | 51.27 | 93.06 |
| D2 | 82128 | 80111 | 33563363 | 418 | 89.15 | 51.91 | 97.54 |
| D3 | 84482 | 80254 | 33697331 | 419 | 88.38 | 52.1 | 95 |
| D4 | 82873 | 80102 | 33266267 | 415 | 87.38 | 50.89 | 96.66 |
| D5 | 83936 | 80396 | 33647938 | 418 | 87.83 | 50.44 | 95.78 |
| D6 | 88575 | 80092 | 33335599 | 416 | 89.33 | 51.29 | 90.42 |
| D7 | 87504 | 80172 | 33666494 | 419 | 89.04 | 51.65 | 91.62 |
| D8 | 86309 | 80133 | 33654327 | 419 | 88.31 | 51.91 | 92.84 |
| D9 | 84329 | 80186 | 33520235 | 418 | 88.43 | 51.63 | 95.09 |
| D10 | 88785 | 80064 | 33628357 | 420 | 89.06 | 51.25 | 90.18 |
| D11 | 82351 | 80100 | 33531900 | 418 | 87.81 | 51.03 | 97.27 |
| D12 | 82885 | 80179 | 33634432 | 419 | 89.52 | 51.23 | 96.74 |
| D13 | 83904 | 80183 | 33499500 | 417 | 89.53 | 51.72 | 95.57 |
| D14 | 84938 | 80296 | 33728896 | 420 | 88.72 | 52.17 | 94.53 |
| D15 | 100522 | 96191 | 40045103 | 416 | 86.1 | 50.77 | 95.69 |
| D16 | 84627 | 80183 | 33706603 | 420 | 88.39 | 50.83 | 94.75 |
| D17 | 83109 | 80086 | 33462848 | 417 | 87.53 | 51.02 | 96.36 |
| D18 | 82196 | 80136 | 33658890 | 420 | 88.93 | 51.8 | 97.49 |
| D19 | 83175 | 80233 | 33391598 | 416 | 89.14 | 51.71 | 96.46 |
| D20 | 85371 | 80215 | 33585177 | 418 | 89.79 | 51.58 | 93.96 |
| D21 | 83954 | 80136 | 33523450 | 418 | 89.3 | 50.8 | 95.45 |
| D22 | 82660 | 80095 | 33461112 | 417 | 89.32 | 51.47 | 96.9 |

Raw Reads refers to sequences that removed low mass and short length; Clean Reads refers to the sequences used for subsequent analysis after removing chimeras; Base refers to the number of bases in the final Clean Reads; AvgLen refers to the average length of Clean Reads; Q20: the percentage of bases with a base mass value greater than 20 (sequencing error rate less than 1%) in Clean Reads; GC (%) indicates the content of GC bases in Clean Reads; Effective (%) indicates the percentage of the number of Clean Reads to that of Raw Reads.
