# Supplementary material for: Changes of saliva microbiota in the onset and after the treatment of diabetes in patients with periodontitis
Source: Aging (Albany NY). 2020 Jul 7;12(13):13090–114. doi: 10.18632/aging.103399 (PMC7377876; doi:10.18632/aging.103399)
Supplement: Supplementary Table 4 [file aging-12-103399-s006..docx]

**Supplementary Table 4. Annotated microbiota to different taxa.**

| Sample Name | OTUs | Species | Genus | Family | Order | Class | Phylum |
| --- | --- | --- | --- | --- | --- | --- | --- |
| A1 | 211 | 106 | 70 | 58 | 32 | 22 | 12 |
| A2 | 159 | 95 | 65 | 53 | 31 | 22 | 12 |
| A3 | 287 | 118 | 93 | 73 | 45 | 29 | 16 |
| A4 | 179 | 99 | 62 | 54 | 33 | 24 | 14 |
| A5 | 228 | 100 | 75 | 61 | 37 | 23 | 12 |
| A6 | 220 | 99 | 65 | 56 | 33 | 23 | 12 |
| A7 | 287 | 123 | 117 | 97 | 57 | 32 | 18 |
| A8 | 242 | 106 | 76 | 56 | 35 | 22 | 12 |
| A9 | 284 | 113 | 101 | 85 | 45 | 25 | 13 |
| A10 | 208 | 100 | 76 | 64 | 39 | 25 | 14 |
| A11 | 215 | 101 | 64 | 61 | 37 | 26 | 14 |
| A12 | 222 | 109 | 72 | 62 | 38 | 25 | 14 |
| A13 | 282 | 117 | 110 | 97 | 58 | 35 | 20 |
| A14 | 252 | 122 | 89 | 70 | 40 | 26 | 14 |
| A15 | 192 | 104 | 70 | 50 | 31 | 22 | 12 |
| A16 | 206 | 106 | 73 | 58 | 32 | 22 | 12 |
| A17 | 236 | 112 | 78 | 62 | 37 | 23 | 13 |
| A18 | 266 | 121 | 86 | 71 | 40 | 24 | 13 |
| A19 | 219 | 105 | 75 | 62 | 36 | 24 | 13 |
| A20 | 170 | 96 | 71 | 63 | 40 | 24 | 14 |
| A21 | 264 | 116 | 83 | 73 | 44 | 26 | 14 |
| A22 | 222 | 112 | 91 | 74 | 45 | 28 | 16 |
| A23 | 178 | 98 | 62 | 51 | 31 | 22 | 13 |
| A24 | 286 | 124 | 93 | 78 | 49 | 31 | 16 |
| A25 | 247 | 111 | 84 | 68 | 41 | 26 | 14 |
| A26 | 287 | 131 | 99 | 77 | 45 | 28 | 17 |
| A27 | 295 | 117 | 95 | 89 | 51 | 32 | 17 |
| A28 | 215 | 104 | 69 | 61 | 38 | 25 | 14 |
| A29 | 244 | 118 | 85 | 69 | 42 | 24 | 13 |
| A30 | 241 | 115 | 79 | 60 | 37 | 23 | 12 |
| A31 | 268 | 113 | 128 | 109 | 64 | 38 | 20 |
| A32 | 135 | 80 | 57 | 52 | 31 | 21 | 12 |
| B1 | 153 | 92 | 60 | 54 | 32 | 21 | 12 |
| B2 | 336 | 150 | 116 | 92 | 54 | 34 | 18 |
| B3 | 309 | 141 | 124 | 102 | 59 | 34 | 16 |
| B4 | 225 | 115 | 79 | 61 | 36 | 25 | 14 |
| B5 | 235 | 119 | 81 | 62 | 34 | 23 | 12 |
| B6 | 325 | 136 | 128 | 109 | 63 | 37 | 19 |
| B7 | 174 | 108 | 69 | 55 | 34 | 22 | 13 |
| B8 | 224 | 120 | 94 | 71 | 41 | 25 | 15 |
| B9 | 179 | 103 | 75 | 65 | 40 | 26 | 14 |
| B10 | 199 | 109 | 72 | 60 | 35 | 24 | 14 |
| B11 | 173 | 100 | 74 | 64 | 36 | 24 | 14 |
| B12 | 137 | 98 | 66 | 51 | 32 | 23 | 13 |
| B13 | 160 | 99 | 67 | 57 | 35 | 23 | 12 |
| B14 | 158 | 102 | 66 | 54 | 32 | 22 | 12 |
| B15 | 199 | 102 | 78 | 76 | 45 | 28 | 15 |
| B16 | 165 | 114 | 74 | 57 | 34 | 23 | 12 |
| B17 | 250 | 119 | 85 | 69 | 41 | 25 | 14 |
| B18 | 236 | 122 | 93 | 71 | 41 | 26 | 15 |
| B19 | 187 | 115 | 78 | 59 | 33 | 23 | 12 |
| B20 | 212 | 117 | 76 | 56 | 32 | 21 | 12 |
| B21 | 150 | 100 | 66 | 52 | 31 | 22 | 12 |
| B22 | 165 | 100 | 68 | 58 | 36 | 23 | 12 |
| B23 | 166 | 99 | 65 | 57 | 32 | 22 | 12 |
| B24 | 225 | 131 | 97 | 73 | 40 | 25 | 15 |
| B25 | 192 | 112 | 79 | 60 | 35 | 22 | 12 |
| B26 | 166 | 107 | 76 | 59 | 37 | 23 | 12 |
| B27 | 251 | 115 | 82 | 69 | 39 | 26 | 14 |
| B28 | 254 | 116 | 87 | 67 | 37 | 23 | 12 |
| B29 | 195 | 106 | 79 | 65 | 37 | 24 | 13 |
| B30 | 137 | 93 | 62 | 51 | 30 | 21 | 11 |
| B31 | 285 | 151 | 108 | 82 | 48 | 30 | 15 |
| C1 | 194 | 110 | 74 | 62 | 36 | 23 | 13 |
| C2 | 163 | 101 | 70 | 60 | 35 | 22 | 12 |
| C3 | 168 | 105 | 73 | 62 | 34 | 23 | 13 |
| C4 | 156 | 88 | 64 | 53 | 32 | 21 | 11 |
| C5 | 179 | 107 | 71 | 62 | 37 | 24 | 13 |
| C6 | 201 | 118 | 87 | 61 | 38 | 23 | 13 |
| C7 | 174 | 105 | 78 | 60 | 35 | 23 | 12 |
| C8 | 246 | 113 | 87 | 72 | 47 | 30 | 17 |
| C9 | 140 | 100 | 68 | 59 | 36 | 23 | 12 |
| C10 | 155 | 102 | 63 | 54 | 32 | 22 | 12 |
| C11 | 203 | 118 | 85 | 65 | 36 | 24 | 14 |
| C12 | 141 | 102 | 78 | 62 | 33 | 23 | 12 |
| C13 | 251 | 127 | 89 | 69 | 41 | 27 | 14 |
| C14 | 209 | 107 | 78 | 64 | 38 | 23 | 13 |
| C15 | 204 | 119 | 86 | 69 | 38 | 22 | 12 |
| C16 | 126 | 99 | 60 | 45 | 27 | 20 | 11 |
| C17 | 169 | 92 | 64 | 57 | 32 | 22 | 11 |
| D1 | 180 | 100 | 69 | 60 | 35 | 22 | 12 |
| D2 | 227 | 121 | 94 | 69 | 41 | 25 | 14 |
| D3 | 108 | 87 | 58 | 48 | 31 | 20 | 11 |
| D4 | 105 | 73 | 54 | 45 | 27 | 21 | 11 |
| D5 | 181 | 108 | 70 | 55 | 34 | 23 | 12 |
| D6 | 232 | 115 | 76 | 60 | 36 | 23 | 13 |
| D7 | 317 | 129 | 106 | 90 | 54 | 34 | 18 |
| D8 | 186 | 104 | 72 | 56 | 31 | 22 | 11 |
| D9 | 237 | 118 | 86 | 65 | 38 | 23 | 13 |
| D10 | 308 | 138 | 103 | 81 | 48 | 24 | 13 |
| D11 | 211 | 113 | 93 | 79 | 46 | 29 | 17 |
| D12 | 216 | 109 | 75 | 61 | 36 | 22 | 12 |
| D13 | 234 | 113 | 75 | 59 | 33 | 23 | 12 |
| D14 | 181 | 104 | 71 | 57 | 33 | 23 | 13 |
| D15 | 208 | 116 | 83 | 63 | 37 | 25 | 14 |
| D16 | 197 | 120 | 98 | 87 | 50 | 30 | 16 |
| D17 | 223 | 114 | 83 | 64 | 37 | 25 | 14 |
| D18 | 115 | 81 | 55 | 46 | 27 | 20 | 11 |
| D19 | 257 | 133 | 102 | 77 | 42 | 28 | 16 |
| D20 | 245 | 118 | 93 | 73 | 41 | 29 | 16 |
| D21 | 218 | 110 | 82 | 69 | 38 | 25 | 15 |
| D22 | 206 | 118 | 84 | 64 | 40 | 25 | 14 |
