# Supplementary material for: Changes of saliva microbiota in the onset and after the treatment of diabetes in patients with periodontitis
Source: Aging (Albany NY). 2020 Jul 7;12(13):13090–114. doi: 10.18632/aging.103399 (PMC7377876; doi:10.18632/aging.103399)
Supplement: Supplementary Table 5 [file aging-12-103399-s002..docx]

**Supplementary Table 5. The periodontitis-associated taxa in each group.**

Genus level:

| A_L&M | wp | B_L&M | wp_XY | C_L&M | wp_XY | C_L&H | wp_XY | C_M&H | wp_XY | D_M&H | wp_XY |
| --- | --- | --- | --- | --- | --- | --- | --- | --- | --- | --- | --- |
| Porphyromonas | 0.037 | Fusobacterium | 0.006 | Rothia | 0.026 | Lautropia | 0.030 | Delftia | 0.045 | Peptostreptococcus | 0.019 |
| Filifactor | 0.037 | Gemella | 0.0027 | Gemella | 0.026 | Filifactor | 0.030 | Desulfobulbus | 0.044 | Meiothermus | 0.045 |
| Parvimonas | 0.041 | Acinetobacter | 0.039 |  |  | Solobacterium | 0.030 | Lactococcus | 0.045 | Barnesiella | 0.045 |
| Streptobacillus | 0.035 |  |  |  |  | Desulfobulbus | 0.012 | Aeromonas | 0.017 |  |  |
| Mycoplasma | 0.022 |  |  |  |  |  |  |  |  |  |  |
| Dialister | 0.034 |  |  |  |  |  |  |  |  |  |  |
| Fretibacterium | 0.0027 |  |  |  |  |  |  |  |  |  |  |
| Schwartzia | 0.0029 |  |  |  |  |  |  |  |  |  |  |
| Pseudoramibacter | 0.034 |  |  |  |  |  |  |  |  |  |  |
| Cetobacterium | 0.038 |  |  |  |  |  |  |  |  |  |  |
| Sphingomonas | 0.047 |  |  |  |  |  |  |  |  |  |  |
| Scardovia | 0.041 |  |  |  |  |  |  |  |  |  |  |
| Simonsiella | 0.0146 |  |  |  |  |  |  |  |  |  |  |
| Murdochiella | 0.017 |  |  |  |  |  |  |  |  |  |  |
| Microbacterium | 0.0075 |  |  |  |  |  |  |  |  |  |  |
| Johnsonella | 0.0078 |  |  |  |  |  |  |  |  |  |  |
| Phenylobacterium | 0.019 |  |  |  |  |  |  |  |  |  |  |
| Gp3 | 0.047 |  |  |  |  |  |  |  |  |  |  |

Species level:

| A_L&M | wp_XY | B_L&M | wp_XY | C_L&M | wp_XY | C_L&H | wp_XY | C_M&H | wp_XY | D_M&H | wp_XY |
| --- | --- | --- | --- | --- | --- | --- | --- | --- | --- | --- | --- |
| *Porphyromonas_gingivalis* | 0.00412 | *Gemella_haemolysans* | 0.0027 | *Rothia_mucilaginosa* | 0.026 | *Eubacterium_sulci* | 0.017 | *Rothia_dentocariosa* | 0.030 | *Peptostreptococcus_stomatis* | 0.019 |
| *Neisseria_oralis* | 0.0089 | *Acinetobacter_nosocomialis* | 0.019 | *Gemella_haemolysans* | 0.026 | *Acinetobacter_johnsonii* | 0.045 | *Dialister_pneumosintes* | 0.030 | *Lactobacillus_sanfranciscensis* | 0.045 |
| *Mycoplasma_faucium* | 0.011 | *Prevotella_fusca* | 0.020 | *Prevotella_dentalis* | 0.0043 | *Lautropia_mirabilis* | 0.030 | *Prevotella_baroniae* | 0.017 | *Treponema_pectinovorum* | 0.045 |
| *Parvimonas_micra* | 0.041 | *Prevotella_buccae* | 0.044 | *Prevotella_buccae* | 0.0087 | *Solobacterium_moorei* | 0.030 | *Prevotella_dentalis* | 0.017 | *Meiothermus_granaticius* | 0.045 |
| *Streptococcus_sobrinus* | 0.0075 |  |  | *Acinetobacter_johnsonii* | 0.028 |  |  | *Treponema_parvum* | 0.045 |  |  |
| *Pseudoramibacter_alactolyticus* | 0.034 |  |  | *Treponema_maltophilum* | 0.041 |  |  | *Prevotella_buccae* | 0.017 |  |  |
| *Cetobacterium_somerae* | 0.038 |  |  |  |  |  |  | *Bacteroides_ovatus* | 0.045 |  |  |
| *Lactobacillus_mucosae* | 0.047 |  |  |  |  |  |  | *Lactococcus_lactis* | 0.045 |  |  |
| *Murdochiella_asaccharolytica* | 0.017 |  |  |  |  |  |  |  |  |  |  |
| *Treponema_parvum* | 0.0078 |  |  |  |  |  |  |  |  |  |  |
| *Olsenella_uli* | 0.036 |  |  |  |  |  |  |  |  |  |  |
| *Actinomyces_timonensis* | 0.0094 |  |  |  |  |  |  |  |  |  |  |
| *Johnsonella_ignava* | 0.0078 |  |  |  |  |  |  |  |  |  |  |
| *Simonsiella_muelleri* | 0.015 |  |  |  |  |  |  |  |  |  |  |
| *Oscillibacter_valericigenes* | 0.047 |  |  |  |  |  |  |  |  |  |  |
| *Haemophilus_sputorum* | 0.049 |  |  |  |  |  |  |  |  |  |  |
| *Fretibacterium_fastidiosum* | 0.0027 |  |  |  |  |  |  |  |  |  |  |
| *Prevotella_enoeca* | 0.015 |  |  |  |  |  |  |  |  |  |  |
| *Schwartzia_succinivorans* | 0.0029 |  |  |  |  |  |  |  |  |  |  |
| *Treponema_maltophilum* | 0.0158 |  |  |  |  |  |  |  |  |  |  |

wp_XY was the p value calculated by the wilcoxon difference test. L, M, and H represent the different severity of periodontitis.
