# Supplementary material for: Changes of saliva microbiota in the onset and after the treatment of diabetes in patients with periodontitis
Source: Aging (Albany NY). 2020 Jul 7;12(13):13090–114. doi: 10.18632/aging.103399 (PMC7377876; doi:10.18632/aging.103399)
Supplement: Supplementary Table 6 [file aging-12-103399-s005..docx]

**Supplementary Table 6. Taxa with significant difference in content between groups**

Genus level:

| Genus | Groups with significant differences |
| --- | --- |
| Acinetobacter | AB_AC_AD_CD |
| Corynebacterium | AB_AD_BC_CD |
| Peptostreptococcus | AB_AD_BC_CD |
| Fusobacterium | AB_AC_AD |
| Stenotrophomonas | AB_AC_AD |
| Streptobacillus | AB_AC_AD |
| Veillonella | AB_AC_AD |
| Blautia | AB_BC_BD |
| Cobetia | AB_BC_BD |
| Nocardia | AB_BC_BD |
| Clostridium_sensu_stricto | AD_BD_CD |
| Faucicola | AD_BD_CD |
| Alloscardovia | AB_AD |
| Gaiella | AB_AD |
| Neisseria | AB_AD |
| Ohtaekwangia | AB_AD |
| Rothia | AB_AD |
| Intrasporangium | AB_AC |
| Moraxella | AB_AC |
| Faecalibacterium | AB_BD |
| Johnsonella | AB_BD |
| Mycoplasma | AB_BD |
| Prevotella | AC_AD |
| Desulfobulbus | AD_BD |
| Methanobrevibacter | AD_BD |
| Vulcaniibacterium | AD_BD |
| Parabacteroides | AC_CD |
| Weissella | BC_BD |
| Aeromonas | BD_CD |
| Peptococcus | BD_CD |
| Lactococcus | BC_CD |
| Aerococcus | AB |
| Atopobium | AB |
| Butyrivibrio | AB |
| Fusicatenibacter | AB |
| Lactobacillus | AB |
| Pantoea | AB |
| Sediminibacterium | AB |
| Eubacterium | AD |
| Filifactor | AD |
| Gemmiger | AD |
| Granulicatella | AD |
| Haemophilus | AD |
| Leptotrichia | AD |
| Anoxybacillus | AC |
| Brevundimonas | AC |
| Delftia | AC |
| Gemmatimonas | AC |
| Nocardioides | AC |
| Streptomyces | AC |
| Actinomyces | BD |
| Burkholderia | BD |
| Phascolarctobacterium | BD |
| Schlegelella | BD |
| Cetobacterium | BC |
| Roseburia | BC |
| Ruminococcus | BC |
| Shuttleworthia | BC |
| Aggregatibacter | CD |
| Bosea | CD |
| Devosia | CD |
| Gp6 | CD |
| Odoribacter | CD |
| Paracoccus | CD |
| Stomatobaculum | CD |

Species level:

| Species | Groups with significant differences |
| --- | --- |
| *Acinetobacter_nosocomialis* | AB\|AC\|AD\|BD |
| *Streptobacillus_moniliformis* | AB\|AC\|AD\|CD |
| *Streptococcus_sobrinus* | AB\|AC\|BD\|CD |
| *Lactobacillus_fermentum* | AB\|BC\|BD\|CD |
| *Campylobacter_rectus* | AB\|AC\|AD |
| *Lactobacillus_salivarius* | AB\|AC\|AD |
| *Leptotrichia_hongkongensis* | AB\|AC\|AD |
| *Pseudomonas_beteli* | AB\|AC\|AD |
| *Veillonella_dispar* | AB\|AC\|AD |
| *Corynebacterium_matruchotii* | AB\|AD\|BC |
| *Peptostreptococcus_stomatis* | AB\|AD\|BC |
| *Dialister_micraerophilus* | AB\|AD\|BC |
| *Alloscardovia_omnicolens* | AB\|AD\|CD |
| *Ohtaekwangia_koreensis* | AB\|AD\|CD |
| *Blautia_wexlerae* | AB\|BC\|BD |
| *Cobetia_amphilecti* | AB\|BC\|BD |
| *Nocardia_coeliaca* | AB\|BC\|BD |
| *Selenomonas_artemidis* | AB\|BC\|BD |
| *Faecalibacterium_prausnitzii* | AB\|BC\|CD |
| *Gaiella_occulta* | AB\|AD |
| *Porphyromonas_gingivalis* | AB\|AD |
| *Prevotella_aurantiaca* | AB\|AD |
| *Rothia_mucilaginosa* | AB\|AD |
| *Johnsonella_ignava* | AB\|BD |
| *Lactobacillus_sanfranciscensis* | AB\|BD |
| *Mycoplasma_faucium* | AB\|BD |
| *Olsenella_uli* | AB\|BC |
| *Actinomyces_georgiae* | AB\|CD |
| *Fusicatenibacter_saccharivorans* | AB\|CD |
| *Faucicola_mancuniensis* | AD\|BD |
| *Vulcaniibacterium_thermophilum* | AC\|BC |
| *Haemophilus_parainfluenzae* | AD\|CD |
| *Weissella_cibaria* | BC\|BD |
| *Actinomyces_timonensis* | AB |
| *Aerococcus_urinaeequi* | AB |
| *Haemophilus_sputorum* | AB |
| *Megasphaera_micronuciformis* | AB |
| *Neisseria_oralis* | AB |
| *Prevotella_oris* | AB |
| *Sediminibacterium_goheungense* | AB |
| *Streptococcus_mutans* | AB |
| *Gemmiger_formicilis* | AD |
| *Granulicatella_adiacens* | AD |
| *Leptotrichia_shahii* | AD |
| *Mycoplasma_lipophilum* | AD |
| *Mycoplasma_spermatophilum* | AD |
| *Prevotella_copri* | AD |
| *Bacteroides_fragilis* | AC |
| *Brevundimonas_nasdae* | AC |
| *Gemmatimonas_aurantiaca* | AC |
| *Prevotella_pallens* | AC |
| *Ralstonia_insidiosa* | AC |
| *Treponema_pectinovorum* | AC |
| *Aggregatibacter_actinomycetemcomitans* | BD |
| *Dialister_invisus* | BD |
| *Lactobacillus_iners* | BD |
| *Mycoplasma_salivarium* | BD |
| *Parabacteroides_goldsteinii* | BD |
| *Peptococcus_niger* | BD |
| *Actinomyces_cardiffensis* | BC |
| *Cetobacterium_somerae* | BC |
| *Eubacterium_yurii_subsp._schtitka* | BC |
| *Lactobacillus_animalis* | BC |
| *Lactococcus_lactis_subsp._tructae* | BC |
| *Roseburia_inulinivorans* | BC |
| *Ruminococcus_bromii* | BC |
| *Shuttleworthia_satelles* | BC |
| *Aggregatibacter_aphrophilus* | CD |
| *Akkermansia_muciniphila* | CD |
| *Anaerococcus_octavius* | CD |
| *Bacteroides_cellulosilyticus* | CD |
| *Parascardovia_denticolens* | CD |
| *Phascolarctobacterium_succinatutens* | CD |
| *Prevotella_nigrescens* | CD |
| *Prevotella_pleuritidis* | CD |
| *Sutterella_stercoricanis* | CD |
| *Thermus_scotoductus* | CD |
