# Supplementary material for: Changes of saliva microbiota in the onset and after the treatment of diabetes in patients with periodontitis
Source: Aging (Albany NY). 2020 Jul 7;12(13):13090–114. doi: 10.18632/aging.103399 (PMC7377876; doi:10.18632/aging.103399)
Supplement: Supplementary Table 7 [file aging-12-103399-s001..docx]

**Supplementary Table 7. Taxa with significant difference in content between groups.**

Genus level:

| AB | BC | BD |
| --- | --- | --- |
| Acinetobacter | Corynebacterium | Blautia |
| Corynebacterium | Peptostreptococcus | Cobetia |
| Peptostreptococcus | Blautia | Nocardia |
| Fusobacterium | Cobetia | Clostridium_sensu_stricto |
| Stenotrophomonas | Nocardia | Faucicola |
| Streptobacillus | Weissella | Faecalibacterium |
| Veillonella | Lactococcus | Johnsonella |
| Blautia | Cetobacterium | Mycoplasma |
| Cobetia | Roseburia | Desulfobulbus |
| Nocardia | Ruminococcus | Methanobrevibacter |
| Alloscardovia | Shuttleworthia | Vulcaniibacterium |
| Gaiella |  | Actinomyces |
| Neisseria |  | Burkholderia |
| Ohtaekwangia |  | Phascolarctobacterium |
| Rothia |  | Schlegelella |
| Intrasporangium |  |  |
| Moraxella |  |  |
| Faecalibacterium |  |  |
| Johnsonella |  |  |
| Mycoplasma |  |  |
| Aerococcus |  |  |
| Atopobium |  |  |
| Butyrivibrio |  |  |
| Fusicatenibacter |  |  |
| Lactobacillus |  |  |
| Pantoea |  |  |
| Sediminibacterium |  |  |

Species level:

| AB | BC | BD |
| --- | --- | --- |
| *Acinetobacter_nosocomialis* | **Lactobacillus_fermentum** | Acinetobacter_nosocomialis |
| *Streptobacillus_moniliformis* | Corynebacterium_matruchotii | Streptococcus_sobrinus |
| *Streptococcus_sobrinus* | Peptostreptococcus_stomatis | **Lactobacillus_fermentum** |
| ***Lactobacillus_fermentum*** | Dialister_micraerophilus | **Blautia_wexlerae** |
| *Campylobacter_rectus* | **Blautia_wexlerae** | Cobetia_amphilecti |
| *Lactobacillus_salivarius* | Cobetia_amphilecti | **Nocardia_coeliaca** |
| *Leptotrichia_hongkongensis* | **Nocardia_coeliaca** | **Selenomonas_artemidis** |
| *Pseudomonas_beteli* | **Selenomonas_artemidis** | Faecalibacterium_prausnitzii |
| *Veillonella_dispar* | Olsenella_uli | Johnsonella_ignava |
| *Corynebacterium_matruchotii* | Weissella_cibaria | Lactobacillus_sanfranciscensis |
| *Peptostreptococcus_stomatis* | Actinomyces_cardiffensis | Mycoplasma_faucium |
| *Dialister_micraerophilus* | Cetobacterium_somerae | Faucicola_mancuniensis |
| *Alloscardovia_omnicolens* | Eubacterium_yurii_subsp._schtitka | Vulcaniibacterium_thermophilum |
| *Ohtaekwangia_koreensis* | Lactobacillus_animalis | Aggregatibacter_actinomycetemcomitans |
| ***Blautia_wexlerae*** | Lactococcus_lactis_subsp._tructae | Dialister_invisus |
| *Cobetia_amphilecti* | Roseburia_inulinivorans | Lactobacillus_iners |
| ***Nocardia_coeliaca*** | Ruminococcus_bromii | Mycoplasma_salivarium |
| ***Selenomonas_artemidis*** | Shuttleworthia_satelles | Parabacteroides_goldsteinii |
| *Faecalibacterium_prausnitzii* |  | Peptococcus_niger |
| *Gaiella_occulta* |  |  |
| *Porphyromonas_gingivalis* |  |  |
| *Prevotella_aurantiaca* |  |  |
| *Rothia_mucilaginosa* |  |  |
| *Johnsonella_ignava* |  |  |
| *Lactobacillus_sanfranciscensis* |  |  |
| *Mycoplasma_faucium* |  |  |
| *Olsenella_uli* |  |  |
| *Actinomyces_georgiae* |  |  |
| *Fusicatenibacter_saccharivorans* |  |  |
| *Actinomyces_timonensis* |  |  |
| *Aerococcus_urinaeequi* |  |  |
| *Haemophilus_sputorum* |  |  |
| *Megasphaera_micronuciformis* |  |  |
| *Neisseria_oralis* |  |  |
| *Prevotella_oris* |  |  |
| *Sediminibacterium_goheungense* |  |  |
| *Streptococcus_mutans* |  |  |
